# Co-inhibition of glutaminolysis and one-carbon metabolism promotes ROS accumulation leading to enhancement of chemotherapeutic efficacy in anaplastic thyroid cancer

**DOI:** 10.1038/s41419-023-06041-2

**Published:** 2023-08-12

**Authors:** Yeseong Hwang, Hyeok Jun Yun, Jae Woong Jeong, Minki Kim, Seyeon Joo, Hae-Kyung Lee, Hang-Seok Chang, Seok-Mo Kim, Sungsoon Fang

**Affiliations:** 1 Graduate School of Medical Science, Brain Korea 21 Project, Yonsei University College of Medicine, Seoul, Korea; 2 Department of Surgery, Thyroid Cancer Center, Institute of Refractory Thyroid Cancer, Gangnam Severance Hospital, Yonsei University College of Medicine, Seoul, Korea; 3 Department of Medicine, Yonsei University College of Medicine, Seoul, Korea; 4 Severance Biomedical Science Institute, Gangnam Severance Hospital, Yonsei University College of Medicine, Seoul, Korea; 5 Chronic Intractable Disease for Systems Medicine Research Center, Yonsei University College of Medicine, Seoul, Korea; 6 Severance Institute for Vascular and Metabolic Research, Yonsei University College of Medicine, Seoul, Korea

**Keywords:** Cancer metabolism, Cancer

## Abstract

Anaplastic thyroid cancer (ATC) is one of the most aggressive tumors with an extremely poor prognosis. Based on the several biological features related to glutamine metabolism in ATC, we hypothesized glutaminolysis inhibition induces cell death in ATC cells. However, glutamine metabolism inhibition triggered cell growth arrest independent of cell death in ATC, suggesting that other signaling pathways avoid glutamine metabolism inhibition-induced stress exist. To investigate the functional mechanism against glutamine metabolism inhibition, we conducted mRNA and ATAC-Sequencing data analysis and found that glutamine deprivation increased ATF4-mediated one-carbon metabolism. When we inhibited PHGDH, the first rate-limiting enzyme for one-carbon metabolism, cell growth arrest was promoted upon glutamine metabolism inhibition by accumulating intracellular ROS. We next observed that the co-inhibition of glutamine and one-carbon metabolism could augment the anticancer effects of drugs used in patients with ATC. Finally, single-cell RNA sequencing analysis revealed that one-carbon metabolism was strengthened through the evolutionary process from PTC to ATC. Collectively, our data demonstrate that one-carbon metabolism has a potential role of modulation of cell fate in metabolic stress and can be a therapeutic target for enhancing antitumor effects in ATC.

## Introduction

Anaplastic thyroid cancer (ATC) is an uncommon, but highly lethal type of dedifferentiated thyroid cancer. Chemotherapy is necessary to prolong survival following surgical resection in ATC. Conventional chemotherapy using doxorubicin or taxanes showed minimal survival benefit and therapies to target tyrosine kinase affecting oncogenic signaling pathway have been developed, including lenvatinib and sorafenib, multi-tyrosine kinase inhibitor. Nonetheless, a combinational approach to overcome minimal effect of monotherapy is still needed in ATC [1–4].

Several studies showed the important roles of glutamine metabolism on ATC tumor environment. It was reported that HER-2 overexpression and frequent β-catenin nuclear localization in ATC, which are associated with increased glutamine metabolism.

Patients with ATC also showed the highest level of glutaminase 1 (GLS1) and glutamate dehydrogenase (GDH) compared to other thyroid cancer types [5–9]. Glutamine is the most abundant amino acid and acts as a precursor of amino acids, proteins, lipids, and nucleotides in humans. Exogenous glutamine is converted to glutamate by GLS, which is again synthesized into α-ketoglutarate by GDH to participate in tricarboxylic acid cycle [10, 11]. This ‘Glutaminolysis’ process is important for tumor growth as it sustains mitochondrial function and reactive oxygen species (ROS) balance. Glutamate is also involved in glutathione (GSH) synthesis, which is a predominant antioxidant enzyme that protects cancer cells from various oxidative stresses such as chemotherapy and radiation [12–15]. Based on the essential roles of glutamine in cancer cell homeostasis, it has been reported that many cancer cells such as lung adenocarcinoma; acute myeloid leukemia; breast; liver; and brain cancer are vulnerable to glutamine deprivation stress, suggesting the crucial role of glutamine in modulating cancer cell survival [16–19].

In contrast, many investigations were also reported that cancer cells exert alternative strategy to survive under glutamine metabolism inhibition. For example, some cancer cells promote glutamine synthesis through glutamine synthetase upregulation or autophagy-mediated glutamine recycling [20, 21]. Another process is to activate one-carbon metabolism pathway. During glutamine deprivation, some cancer cells particularly uptake serine, which is a major donor of one-carbon metabolism. One-carbon metabolism including folate and methionine cycle is important physiological process to overcome nutrition deficiency through diverse outputs such as amino acids, lipids and ROS imbalance through GSH synthesis. Thus, one-carbon metabolism is crucial for maintaining tumorigenesis upon glutamine metabolism inhibition-induced nutrient reprogramming [22–26].

Based on strong relationship between glutamine metabolism and biological features of ATC, we hypothesized ATC are vulnerable to glutaminolysis inhibition. Unexpectedly, we found that ATC cells are resistant to cell death owing to the induction of one-carbon metabolism by controlling redox balance during glutamine metabolism inhibition. We also confirmed that combined inhibition of glutamine and one-carbon metabolism is sufficient to reduce cell proliferation more than treated alone and increase the efficacy of lenvatinib and sorafenib, a multitarget tyrosine kinase inhibitor for patients with ATC. In addition, we identified ATC might intensify one-carbon metabolism following evolutionary route from papillary thyroid cancer (PTC) in single-cell analysis. Our data suggest possibility that ATC employs one-carbon metabolism to rapidly adapt to diverse stresses such as metabolic stress and chemotherapy and provide therapeutic target for ATC by inhibiting one-carbon metabolism.

## Results

### Glutaminolysis is highly upregulated in ATC patients

**Fig. 1:**
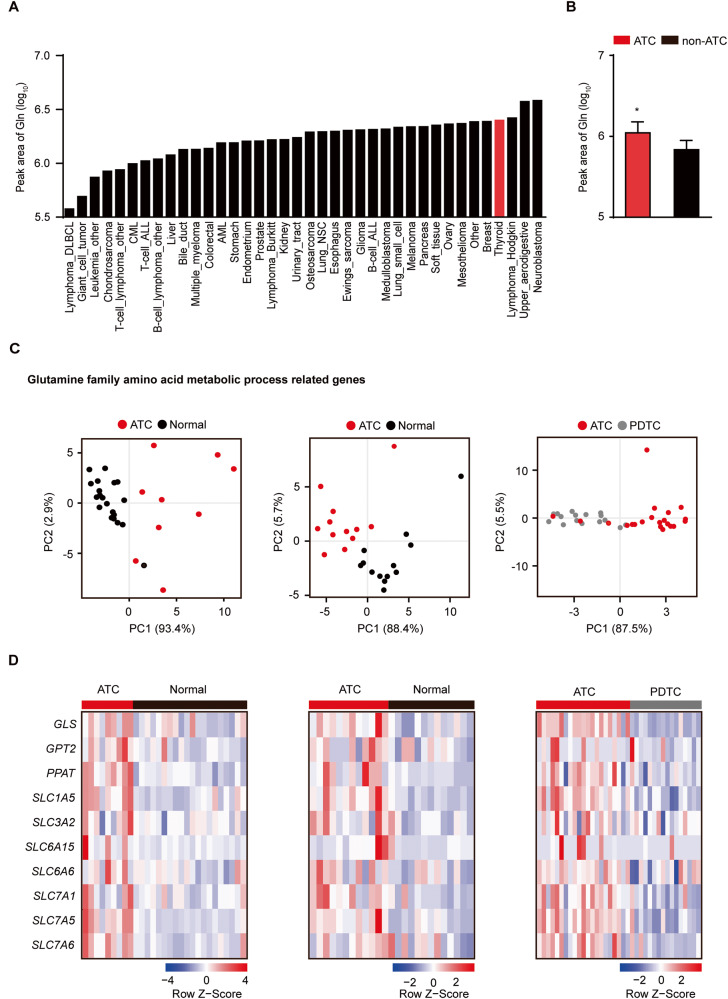
ATC patients exhibit glutaminolysis enhancement. **A** Bar graph represents peak area of glutamine level in diverse cancer types using CCLE metabolomic data. **B** The bar graph shows comparison of glutamine concentration between ATC (8505 C, CAL62, BHT101, 8305 C) and non-ATC (BCPAP, FTC238, FTC133, ML1, TT2609C02) cell lines as revealed by CCLE metabolomic data. **C** Principal component analysis shows separation of glutamine family amino acid metabolic process genes in GSE datasets (GSE29265; ATC patients (*n* = 9)/normal subjects (*n* = 20), GSE65144; ATC patients (*n* = 12)/normal subjects (*n* = 13), GSE76039; PDTC patients (*n* = 16)/ATC patients (*n* = 21)). **D** Heatmap represents comparison of glutaminolysis-related genes. The groups are indicated in **C**. Color scale means each gene expression level. Statistical comparisons were performed using two-tailed unpaired Student’s *t* test. (*P < 0.05).

We first compared glutamine concentration in diverse cancer types using the Cancer Cell Line Encyclopedia (CCLE) [27]. Thyroid cancer cells had high level of intracellular glutamine (Fig. 1A). Interestingly, we observed glutamine level in ATC cell lines was higher than non-ATC groups [28], indicating that glutamine may be an important factor for ATC cell metabolism (Fig. 1B). Using Gene Expression Omnibus (GEO) data, we compared gene expression levels related to glutamine between ATC patients and normal subjects or patients with poorly differentiated thyroid cancer (PDTC), who have an intermediate spectrum of differentiation leading to a longer survival rate compared to patients with ATC [29]. Principal component analysis showed patients with ATC had distinct glutamine family amino acid metabolic process-related genes from normal or patients with PDTC (Fig. 1C). In addition, the expression of glutaminolysis-related genes, such as *GLS, GPT2, and SLC1A5* was highly upregulated in patients with ATC (Fig. 1D). Taken together, these data demonstrate patients with ATC have a high rate of glutaminolysis, which may indicate a high dependency of ATC on glutamine.

### Glutamine metabolism inhibition promotes one-carbon metabolism in ATC

**Fig. 2:**
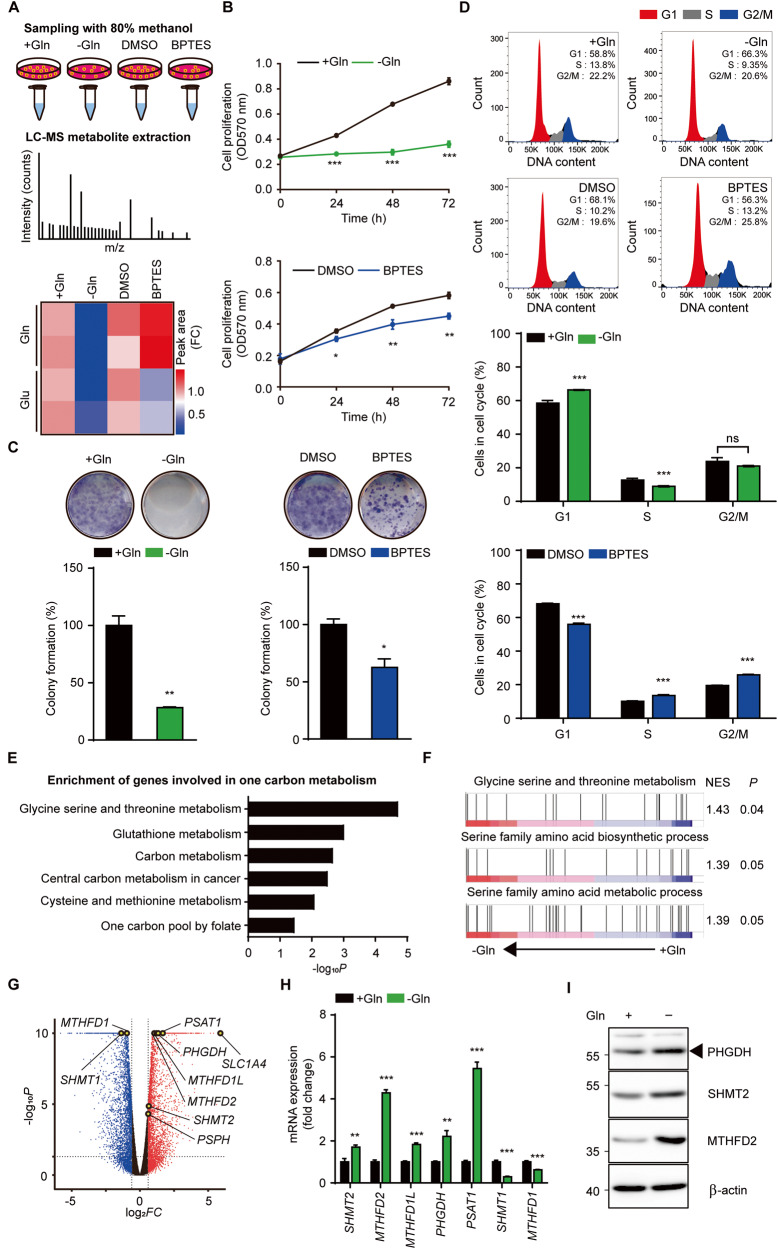
Glutamine metabolism inhibition increases one-carbon metabolism in ATC. **A** Workflow depicts sample processing and LC-MS analysis treated with glutamine deprivation medium or BPTES (10 μM) for 72 h (top). Heatmap shows peak area of intracellular glutamate and glutamine in indicated groups. The scale means fold change of peak area of metabolite (bottom). The images of workflow are acquired from MOTIFOLIO site. **B** 8505 C ATC cells were treated with glutamine deprivation or BPTES (10 μM) for 72 h. Cell proliferation was measured by MTT. **C** Cells were treated with glutamine deprivation or BPTES (10 μM) for 10 days. Colony formation was stained with crystal violet solution and dissolved in methanol for quantification. **D** Cells were treated with glutamine deprivation or BPTES (10 μM) for 24 h. Cell cycle was analyzed by Propidium Iodide staining in flow cytometry (top). Bar graph shows percentage of cells in each cell cycle fraction (bottom). **E** KEGG analysis of DEGs shows one-carbon metabolism-related pathways were increased for 24 h glutamine deprivation. **F** GSEA plots show gene enrichment pattern of glycine serine and threonine metabolism, serine family amino acid biosynthetic and metabolic process were increased for 24 h glutamine deprivation. **G** Volcano plot represents up and down of one-carbon metabolism-related genes for 24 h glutamine deprivation (|log_2_ FC| ≥ 0.5) *P < 0.05). **H** Cells were treated with glutamine deprivation for 24 h. *SHMT2*, *MTHFD2*, *MTHFD1L, PHGDH, PSAT1*, *SHMT1*, *MTHFD1* mRNA expressions were assessed by real-time RT-PCR. **I** Western blots show PHGDH, SHMT2, MTHFD2, β-actin expressions for 24 h glutamine deprivation. The number left on the immunoblot images indicates the protein size of the immunoband measured for this analysis. Data are expressed as the mean S.D. of three independent experiments (*n* = 3). Statistical comparisons were performed using two-tailed Student’s *t* test. (*P < 0.05; **P < 0.01; ***P < 0.001; ns, not significant).

Based on positive correlation of ATC with glutaminolysis, we hypothesized aberrant glutamine metabolism through glutamine withdrawal or GLS1 inhibition with BPTES could lead to significant cellular stress and subsequent cell death. We observed glutamate level to confirm whether these two approaches indeed suppress glutaminolysis using LC-MS metabolite analysis. Glutamine deprivation reduced intracellular glutamine levels and BPTES showed opposite effect. However, glutamine deprivation and BPTES decreased cellular glutamate level, suggesting that these two approaches are interchangeable in the case of glutaminolysis impairment (Fig. 2A) [30, 31]. Glutamine metabolism inhibition with glutamine deprivation or BPTES markedly reduced cell growth and the number of colonies stained with crystal violet in 8505 C (Fig. 2B, C). Nonetheless, there was no change in apoptotic markers or LDH release from damaged cells upon inhibition of glutamine metabolism, which is consistent with another SNU-80 ATC cell (Fig. S1A–C). Glutamine deprivation or BPTES-treated 8505 C stained with Hoechst 33342 also showed intact nuclei, indicating that inhibition of glutaminolysis do not elicit significant DNA damage (Fig. S2A, B). To determine why inhibition of glutaminolysis reduces cell proliferation, we investigated cell cycle progression in 8505 C [10, 32]. While glutamine deprivation triggered cell cycle arrest by reducing S-phase and enhancing G1-phase fraction, BPTES arrested cell cycle by increasing S-phase and G2/M-phase fraction (Fig. 2D). As inhibition of glutamine metabolism leads to cell growth impairment but not cell death, we hypothesized certain pathways to avoid inhibition of glutamine metabolism exist in 8505 C. To identify this signaling pathway, we used differentially expressed genes (DEGs) from cells cultured in glutamine-full and glutamine-free medium. We confirmed glutamine deprivation-induced distinct differences in one-carbon metabolism-related pathways, contributing to tumorigenic effects in cancer cells (Fig. 2E) [24–26]. Several pathways-related to serine metabolism were also enriched in glutamine-deprived cells based on gene set enrichment analysis (GSEA) (Fig. 2F). We found glutamine deprivation increased mitochondrial genes such as *SHMT2*, *MTHFD2*, *MTHFD1L* and cytosolic genes such as *PHGDH*, *PSAT1*, *PSPH*. In contrast, cytosolic genes such as *SHMT1* and *MTHFD1* were downregulated, consistent with the results of mRNA level (Fig. 2G, H). Glutamine deprivation also increased protein levels of PHGDH, SHMT2 and MTHFD2 (Fig. 2I). Our data demonstrate glutamine deprivation upregulates one-carbon metabolism pathway, which might be alternative strategy to endure glutamine deprivation-induced metabolic changes in ATC cells.

### ATF4 is a dominant regulator of one-carbon metabolism during glutamine deprivation

**Fig. 3:**
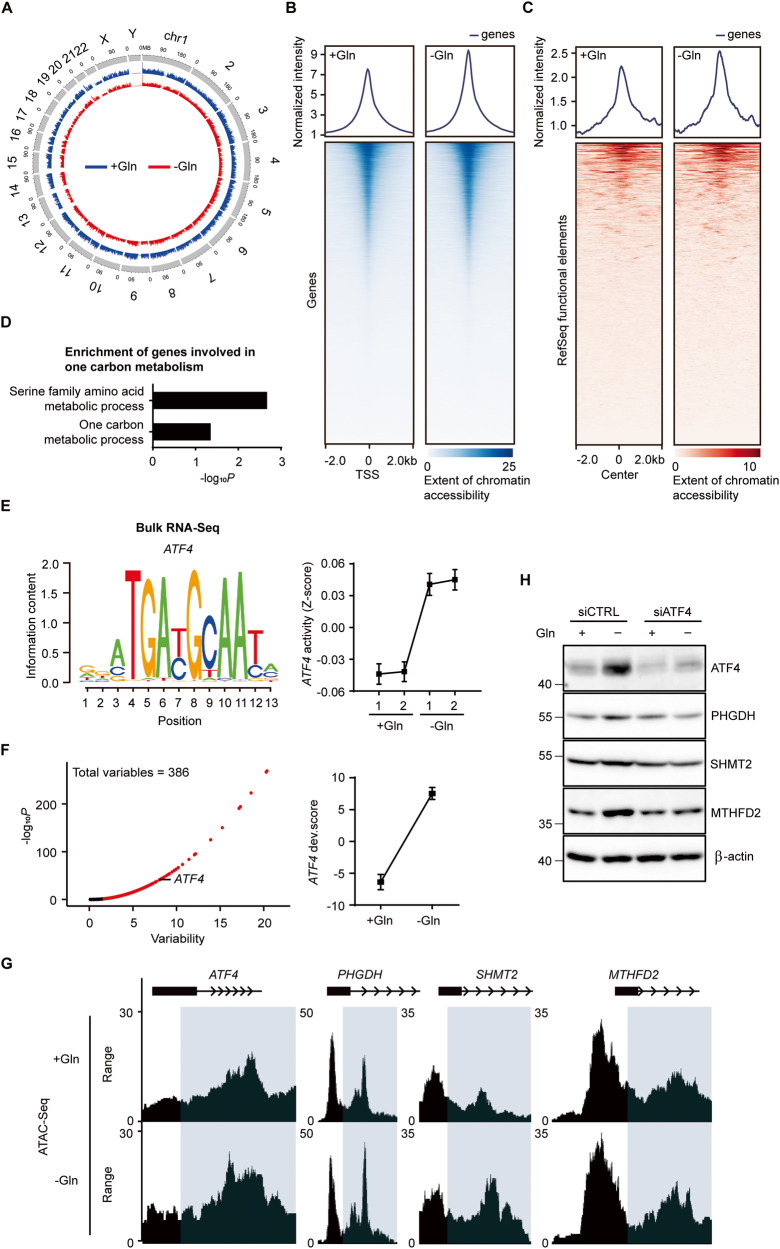
ATF4 upregulates one-carbon metabolism under glutamine deprivation. **A** ShinyCircos graph depicts the genome-wide chromatin accessibility on chromosomes in glutamine-full and glutamine-free medium cultured cells for 24 h. **B** The Heatmap shows ATAC-Seq peaks on the transcription start site aligned to their centre ± 2 kb. Color intensity shows extent of chromatin accessibility. **C** Heatmap of ATAC-Seq peaks based on RefSeq functional elements aligned to their centre ± 2 kb. Color intensity means extent of chromatin accessibility. **D** Gene ontology analysis shows enrichment of biological processes related to one-carbon metabolism in glutamine deprivation cells. *p*-value is based on the binomial test (*P < 0.05). **E** ISMARA motif analysis shows significant activity change of *ATF4* under glutamine-free medium cultured cells for 24 h. *Z* score means motif activity of *ATF4*. **F** *ATF4* motif activity during glutamine deprivation for 24 h was significantly upregulated as revealed by chromVAR. **G** GBiB shows a comparison of ATAC-Seq peak signals from indicated genes in glutamine-full and glutamine-free medium cultured cells for 24 h. **H** Immunoblot assay of ATF4, PHGDH, SHMT2, MTHFD2, β-actin in siCTRL and siATF4-transfected cells under glutamine deprivation for 24 h. The number left on the immunoblot images indicates the protein size of the immunoband measured for this analysis.

Next, we determined whether gene expression changes via glutamine deprivation are associated with chromatin accessibility, as chromatin modification is a rapid, reversible event that allows cells to adapt to environmental stress such as nutrient deprivation [33]. Based on Assays for Transposase Accessible Chromatin with high-throughput sequencing (ATAC-Seq) data, we first determined overall peak signals of chromosomes and confirmed no significant change in differential peaks (Fig. 3A). However, glutamine deprivation increased chromatin accessibility at the transcription start site and RefSeq functional elements (Fig. 3B, C). When we next identified regions with modified chromatin accessibility through gene ontology analysis, enrichment of biological processes associated with one-carbon metabolism was increased in glutamine deprivation cells (Fig. 3D). ATF4 upregulates one-carbon metabolism gene due to transcriptional dysregulation [34–36]. As we predicted *ATF4* activity would increase in mRNA-seq analysis under glutamine deprivation (Fig. 3E), we confirmed *ATF4* activity under glutamine deprivation-induced metabolic reprogramming in ATAC-Seq data. Consistent with Integrated Motif Activity Response Analysis (ISMARA), *ATF4* was considered significant in 386 variables, and the developmental score was increased in glutamine deprivation (Fig. 3F). We also observed ATAC-Seq peaks for *ATF4* and downstream factors such as *PHGDH*, *SHMT2*, and *MTHFD2* were high in the absence of glutamine through Genome Browser in a Box (GBiB) (Fig. 3G). Our in vitro experiment directly showed that enhanced one-carbon metabolism-related protein through glutamine deprivation was abrogated by genetic inhibition of ATF4 (Fig. 3H, S3), indicating that ATF4 is a critical transcriptional regulator of one-carbon metabolism under glutamine deprivation in ATC cells.

### Blocking one-carbon metabolism promotes ROS accumulation leading to cell proliferation arrest

**Fig. 4:**
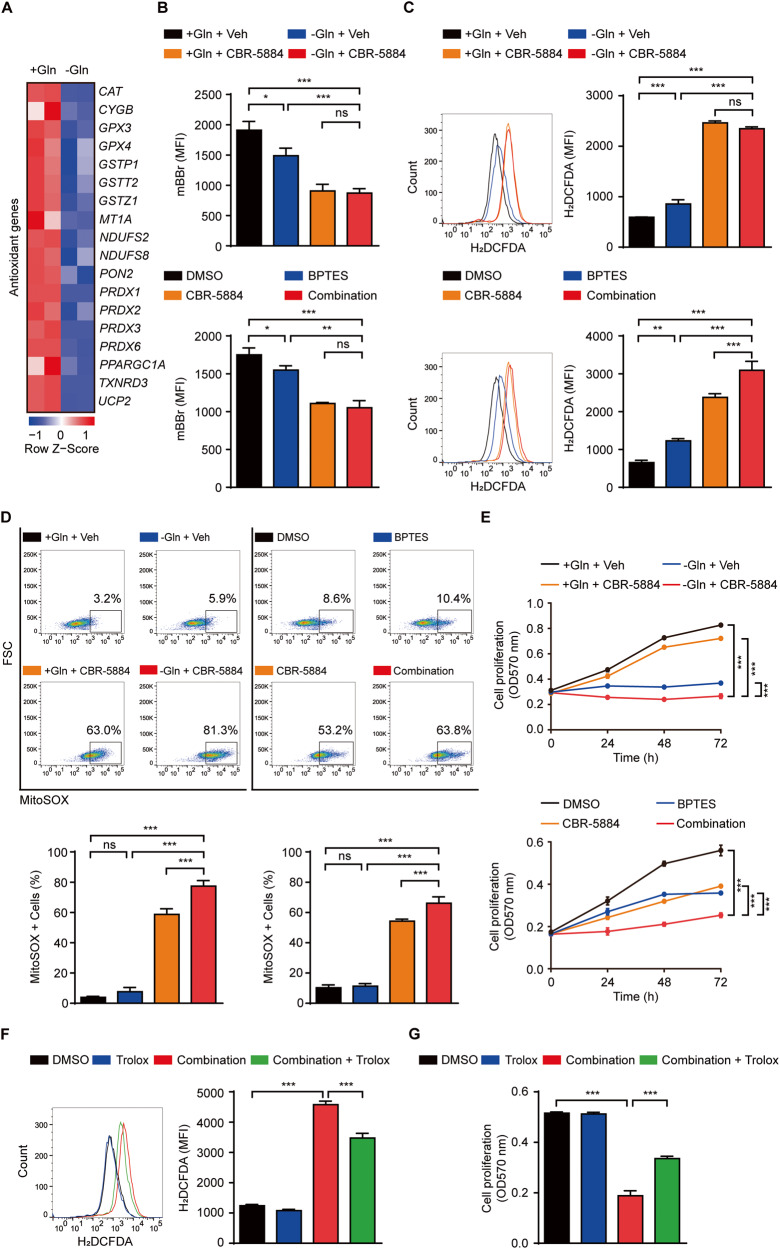
Impairment of one-carbon metabolism decreases cell proliferation by accumulating ROS. **A** Heatmap shows decreased level of antioxidant-related genes during glutamine deprivation for 24 h in 8505 C ATC cells (|FC| ≥ 1.4, *P < 0.05). Row *Z* score represents transformed FPKM values. **B** Cells were treated with indicated groups (glutamine-free medium, BPTES; 10 μM, CBR-5884; 60 μM) for 24 h. The bar graph shows the detection of total GSH levels in indicated groups by mBBr staining (10 μM) in flow cytometry. **C** Cells were treated with indicated groups (glutamine-free medium, BPTES; 10 μM, CBR-5884; 60 μM) for 24 h. Bar graph shows intracellular ROS level in indicated groups by H_2_DCFDA staining (10 μM) in flow cytometry. Representative histograms are shown (left panel). **D** Cells were treated with indicated groups (glutamine-free medium, BPTES; 10 μM, CBR-5884; 60 μM) for 24 h. Bar graph shows mitochondrial ROS accumulation in indicated groups by MitoSOX^TM^ Red staining (5 μM) in flow cytometry. Representative plots are shown (upper panel). **E** Cells were treated with indicated groups (glutamine-free medium, BPTES; 10 μM, CBR-5884; 60 μM) for 72 h. Cell proliferation was measured by MTT. **F** Cells were treated with indicated groups for 24 h (BPTES; 10 μM, CBR-5884; 60 μM, Trolox; 25 μM). The bar graph shows intracellular ROS level in indicated groups by H_2_DCFDA staining (10 μM) in flow cytometry. Representative histograms are shown (left panel). **G** Cells were treated with indicated groups for 72 h (BPTES; 10uM, CBR-5884; 60 μM, Trolox; 25 μM). Cell proliferation was measured by MTT. Data are expressed as the mean S.D. of three independent experiments (*n* = 3). Statistical comparisons were performed using ANOVA followed by Tukey’s multiple comparison test. (*P < 0.05; **P < 0.01; ***P < 0.001; ns, not significant).

Given that one-carbon metabolism controls redox balance, which protects cancer cells from growth arrest during diverse stresses [23, 37–39], we next studied one-carbon metabolism and its causal relationship with resistance to glutamine metabolism inhibition. We first identified decreased levels of antioxidant-related DEGs such as *CAT*, *TXNRD3*, and *GPX4* and total GSH levels that determine cellular redox potential were reduced by monobromobimane (mBBr) staining in the absence of glutamine, suggesting that glutamine metabolism inhibition only triggers oxidative stress (Fig. 4A, B). To identify whether the inhibition of one-carbon metabolism exerts additive effects on GSH reduction, we used CBR-5884 to inhibit PHGDH, the first enzyme for initiating one-carbon metabolism [40]. CBR-5884 promoted total GSH reduction with inhibition of glutamine metabolism (Fig. 4B). As reduced GSH levels cause cellular ROS increase [41, 42], we compared intracellular ROS levels as revealed by H_2_DCFDA staining. Inhibition of glutamine metabolism elevated cellular ROS, which was strongly accumulated by CBR-5884, supporting the pivotal role of one-carbon metabolism in the regulation of redox balance (Fig. 4C, S4A). We next studied whether inhibition of one-carbon metabolism increases mitochondrial ROS as mitochondrial one-carbon metabolism genes *SHMT2*, *MTHFD2,* and *MTHFD1L* were increased during glutamine deprivation (Fig. 2G, H). Although inhibition of glutamine metabolism did not increase mitochondrial ROS, inhibition of one-carbon metabolism increased mitochondrial ROS, which was aggravated upon glutamine and one-carbon metabolism inhibition (Fig. 4D). Moreover, co-inhibition of glutamine and one-carbon metabolism blocked cell growth more than treated alone (Fig. 4E, S4B), indicating that ATC cells employ one-carbon metabolism pathway to endure metabolic stress from glutamine metabolism inhibition. This compromised cell proliferation was consistently represented in the one-carbon metabolism inhibitors SHMT1/2 and MTHFD2 (Fig. S5A, B). To confirm possible impairment of proliferation due to ROS overload, we treated cells with antioxidant Trolox [43, 44]. When Trolox reduced ROS levels, cell proliferation was rescued in combination (BPTES+ CBR-5884) treatment (Fig. 4F, G). Our data demonstrate one-carbon metabolism is a compensatory pathway to avoid glutamine metabolism inhibition, and ROS overburden through glutamine and one-carbon metabolism inhibition is a key event in suppressing tumorigenesis.

### Co-inhibition of glutamine and one-carbon metabolism synergistically increases chemotherapy efficacy

**Fig. 5:**
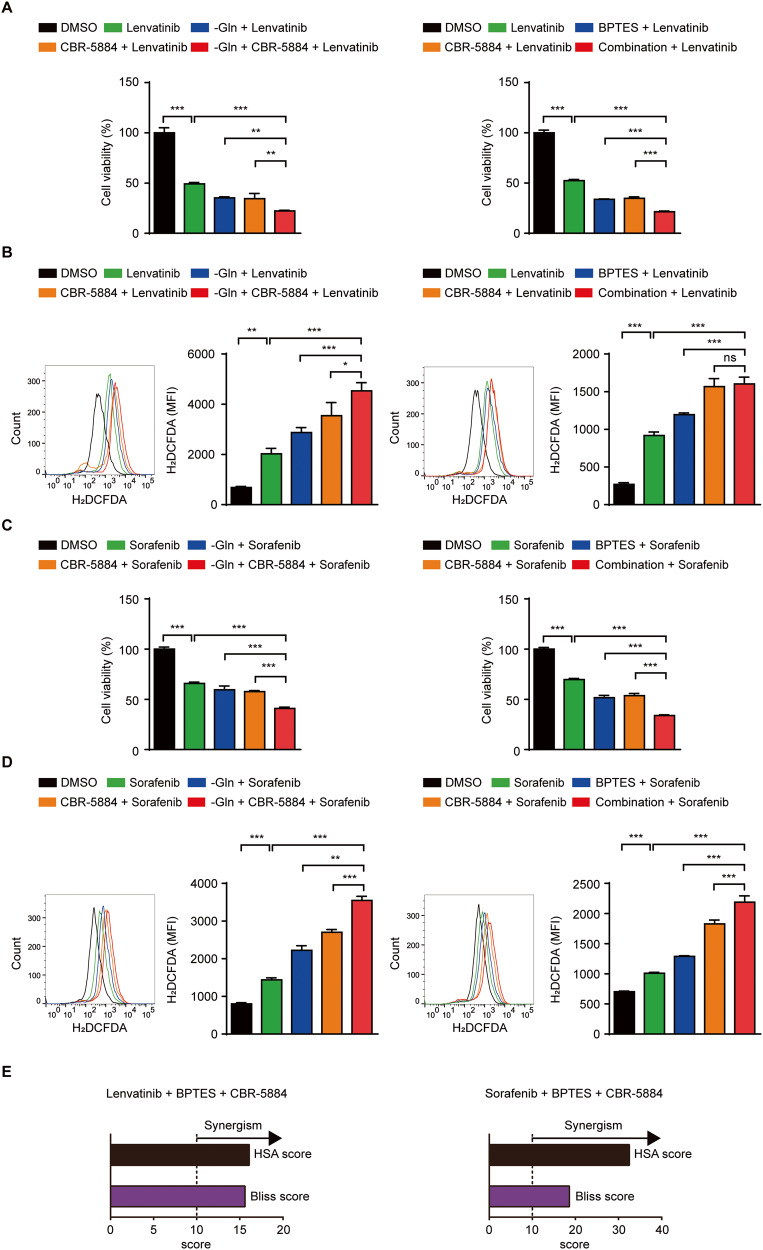
Combinational inhibition of glutamine and one-carbon metabolism synergistically enhances chemotherapy efficacy. **A** Cells were treated with indicated groups (glutamine-free medium, lenvatinib; 50 μM; BPTES; 10 μM, CBR-5884; 60 μM) for 48 h. Cell viability was measured by MTT. **B** Cells were treated with indicated groups (glutamine-free medium, lenvatinib; 50 μM; BPTES; 10 μM, CBR-5884; 60 μM) for 9 h. Bar graph shows intracellular ROS level in indicated groups by H_2_DCFDA staining (10 μM) in flow cytometry. Representative histograms are shown (left panel). **C** Cells were treated with indicated groups (glutamine-free medium, sorafenib; 10uM; BPTES; 10 μM, CBR-5884; 60 μM) for 48 h. Cell viability was measured by MTT. **D** Cells were treated with indicated groups (glutamine-free medium, sorafenib; 10 μM; BPTES; 10 μM, CBR-5884; 60 μM) for 9 h. Bar graph shows intracellular ROS level in indicated groups by H_2_DCFDA staining (10 μM) in flow cytometry. Representative histograms are shown (left panel). **E** Cells were treated with indicated single drug (lenvatinib; 50 μM; sorafenib; 10 μM; BPTES; 10 μM, CBR-5884; 60 μM) for 48 h. The synergistic effects of multiple drugs were confirmed using HSA and Bliss model. Data are expressed as the mean S.D. of three independent experiments (*n* = 3). Statistical comparisons were performed using ANOVA followed by Tukey’s multiple comparison test. (*P < 0.05; **P < 0.01; ***P < 0.001; ns, not significant).

Based on our results that ROS overload disrupts cell tumorigenicity, we studied clinical synergy of co-inhibitory pathways with lenvatinib and sorafenib, which block multi-tyrosine kinases such as Vascular Endothelial Growth Factor Receptor (VEGFR) and Platelet Derived Growth Factor Receptor (PDGFR) in patients with ATC [2, 3]. First, IC_50_ values of two drugs were calculated (Fig. S6A, C), and confirmed the inhibition of glutaminolysis only enhanced drug efficacy (Fig. S6B, D). In addition, glutamine metabolism inhibition contributed to drug-induced ROS accumulation (Fig. S6E, F). We observed co-inhibition of glutamine and one-carbon metabolism most strongly suppressed cell viability and enhanced ROS levels after treatment with chemotherapeutic agents (Fig. 5A–D, and S7A, B). Finally, Highest single agent (HSA) and Bliss reference model showed that multi-drug combinations elicited synergy effect in lenvatinib and sorafenib-treated ATC cells (Fig. 5E, S7C). In conclusion, our data imply combined targeting of glutaminolysis and one-carbon metabolism could be efficient in enhancing chemotherapy efficacy by accumulating ROS for ATC.

### ATC displays enhanced serine and one-carbon metabolism dependency compared to PTC

**Fig. 6:**
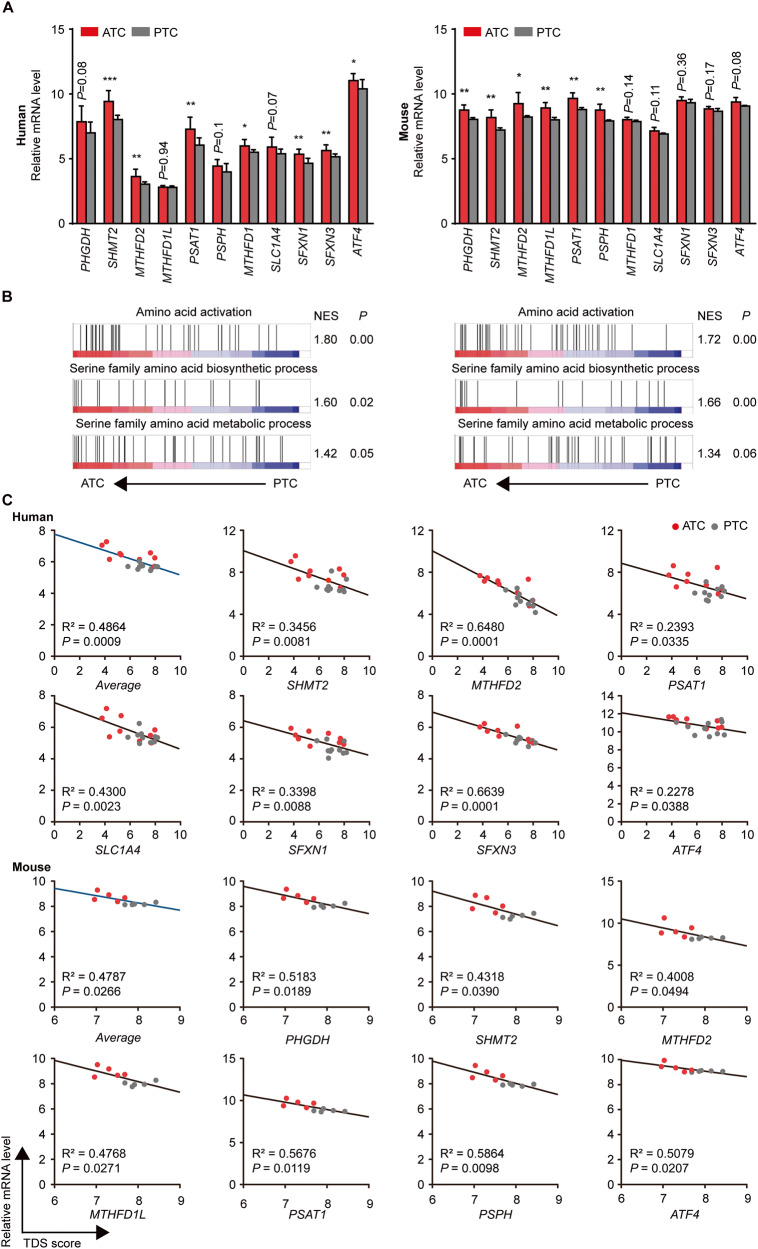
One-carbon metabolism in ATC is more active than PTC. **A** Bar graphs show mRNA levels of one-carbon metabolism-related genes in human and mouse GSE datasets (GSE29265; ATC patients (*n* = 9)/PTC patients (*n* = 10), GSE55933; TPO-creER;Braf^V600E^/p53^−/−^ ATC mice (*n* = 5)/TPO-creER;Braf^V600E^ PTC mice (*n* = 5)). **B** GSEA plots show gene enrichment pattern of amino acid activation, serine family amino acid biosynthetic and metabolic process were increased in human and mouse ATC compared with PTC. **C** The average and several one-carbon metabolism genes (*y* axis) show a negative correlation with TDS score (*x* axis) in ATC and PTC of human and mouse. The black lines indicate simple linear regression. Data are expressed as the mean S.D. Statistical comparisons were performed using a two-tailed Student’s *t* test. (*P < 0.05; **P < 0.01; ***P < 0.001).

One-carbon metabolism is critical in tumorigenicity against glutamine metabolic changes and chemotherapy in ATC. It is also well known that PTC histologically progresses to ATC by dedifferentiation [45, 46]. To explore divergent characteristics associated with one-carbon metabolism between ATC and PTC, we compared levels of total 11 one-carbon metabolism genes in human and mouse GEO datasets. mRNA levels of several one-carbon metabolism-related genes such as *SHMT2*, *MTHFD2*, *PSAT1,* and *MTHFD1* in ATC were higher than those in PTC patients. Tamoxifen-treated TPO-creER;Braf^V600E^/p53^-/-^ transgenic mice, which exhibit ATC features including shortened survival and histologic morphology, also revealed high level of one-carbon metabolism genes such as *PHGDH*, *SHMT2*, *MTHFD2*, *MTHFD1L*, *PSAT1,* and *PSPH* compared to PTC mice despite different tendency with human (Fig. 6A). We also confirmed several pathways including amino acid activation, serine family amino acid biosynthetic and metabolic process of ATC were enriched compared with PTC in human and mouse, suggesting ATC might increase serine and one-carbon metabolism dependency more than PTC (Fig. 6B). We next analyzed correlation between one-carbon metabolism genes and the average of total 16 thyroid differentiation score genes (hereafter TDS score), which illustrates thyroid function of PTC and significantly decline in ATC [47]. Although several genes showed no significant correlation, we identified average of total 11 one-carbon metabolism genes exhibited negative correlation with TDS score in human and mouse, again supporting strong correlation between ATC and one-carbon metabolism (Fig. 6C, S8). Taken together, our data represent ATC might develop serine and one-carbon metabolism availability during progression from PTC, which contributes to defense mechanism against antitumor stress.

### One-carbon metabolism may seem to be intensified according to evolutionary path from PTC to ATC

**Fig. 7:**
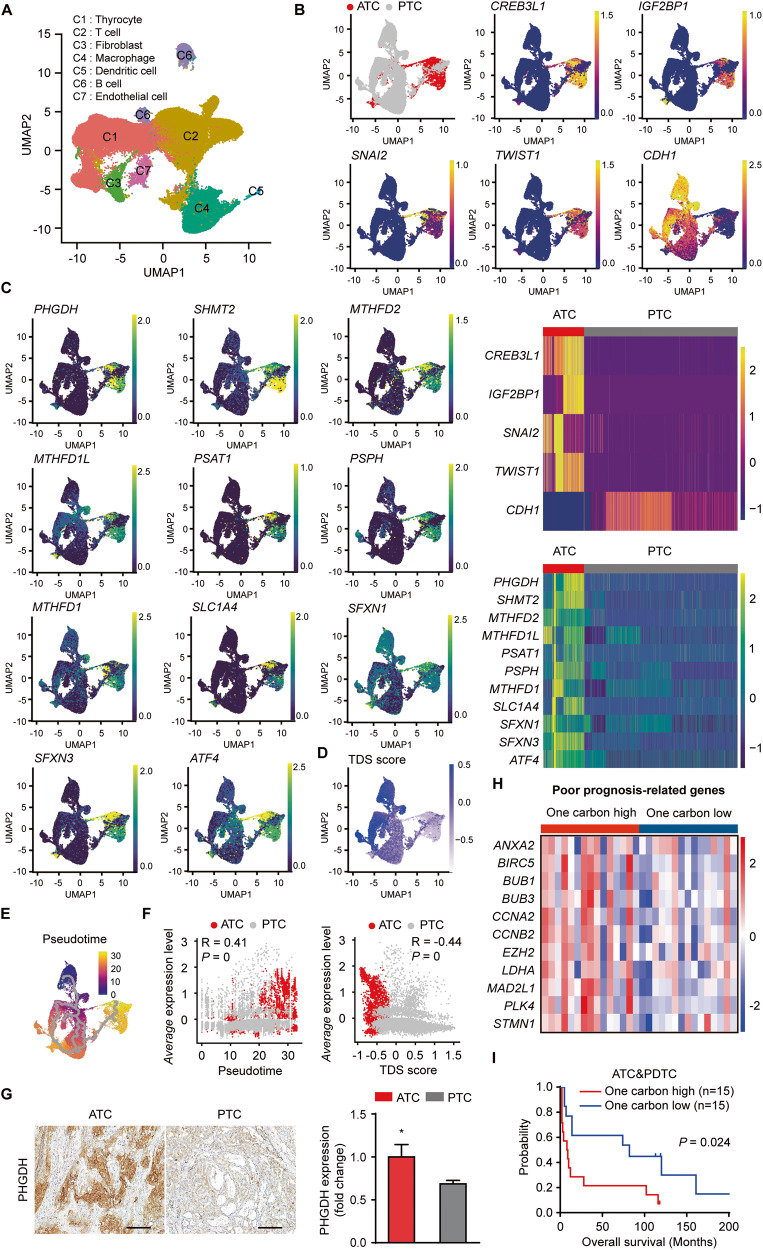
One-carbon metabolism might be strengthened following evolution routes from PTC to ATC. **A** UMAP shows cell types in integrated ATC (*n* = 5) and PTC (*n* = 7) patients. The GEO datasets of ATC and PTC are from GSE148673 and GSE184362, respectively. **B** UMAP of integrated thyrocytes shows division of ATC and PTC clusters through several markers. The magma color scale represents the expression level in each gene (top). Heatmap shows the expression level of left panel 5 genes. The color scale means expression level of each gene. These 5 genes are statistically significant (bottom). (***P < 0.001) **C** UMAP of integrated thyrocytes represent the comparison of total 11 one-carbon metabolism-related genes such as *PHGDH*, *SHMT2*, *MTHFD2*, *ATF4* between ATC and PTC clusters. The viridis scale indicates expression level in each gene (left panel). Heatmap exhibits the expression level of left panel 11 genes. The color scale represents expression level of each gene. These 11 genes are statistically significant (right panel). (***P < 0.001) **D** UMAP of integrated thyrocytes shows decreased TDS score from PTC to ATC. The blue scale indicates expression level in TDS score genes. **E** Monocle3 shows colored scale and gray line representing the pseudotime and inferred trajectory respectively from PTC to ATC progression. **F** Scatterplot represents positive correlation between average expression level of total 11 one-carbon metabolism genes (*y* axis) and pseudotime (*x* axis) in ATC and PTC patients (left panel). Scatterplot exhibits negative correlation between average expression level of total 11 one-carbon metabolism genes (*y* axis) and TDS score (*x* axis) in ATC and PTC patients (right panel). **G** IHC staining shows PHGDH expression in ATC and PTC patients (ATC patients=3, PTC patients = 4). Scale bar = 100 μm. **H** Heatmap shows one-carbon metabolism high groups exhibit enrichment pattern of poor prognosis genes associated with thyroid cancer. The color scale represents the expression level of each gene. **I** Kaplan–Meier survival curve shows the overall survival in aggressive thyroid cancer patients separated by one-carbon metabolism-related genes high (*n* = 15) and low (*n* = 15) groups. The dataset of ATC and PTC patients is from GSE76039 (ATC (*n* = 18)/PDTC patients (*n* = 17)). Statistical comparisons were performed using ‘FindMarkers’ in R software (**B**, **C**). Data are presented as the means ± SD. statistical comparisons were performed using a two-tailed unpaired Student’s *t* test in **G**. (*P < 0.05).

**Fig. 8:**
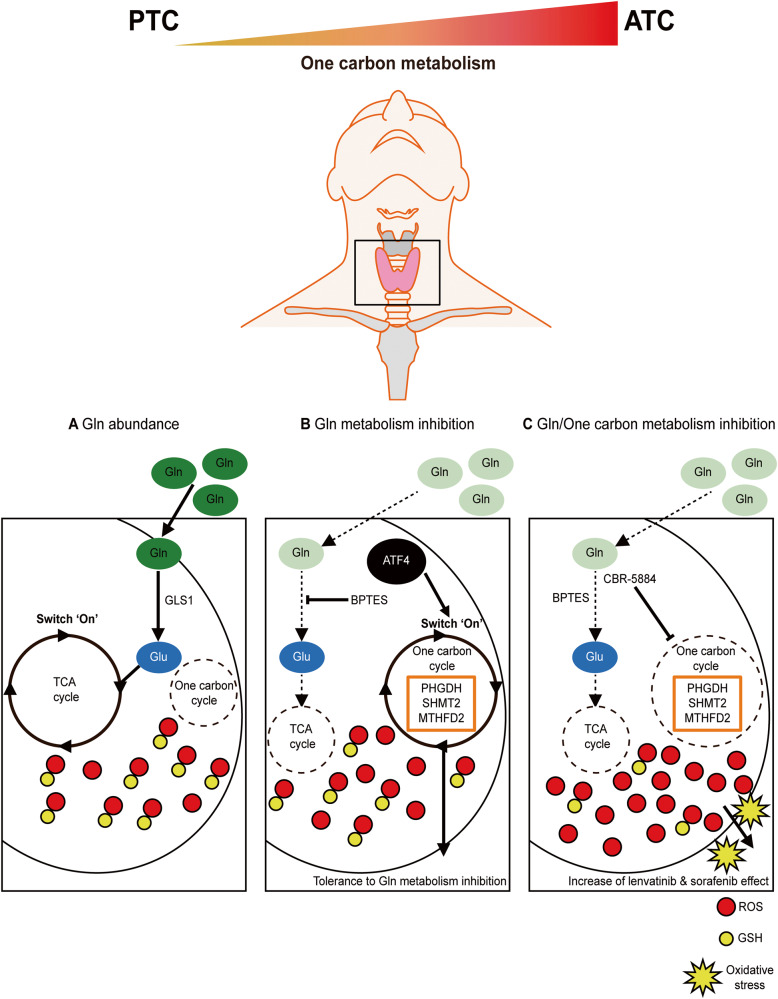
Schematic diagram of the molecular characteristics to enhance therapeutic effects for ATC. One-carbon metabolism was gradually more active during progression from PTC to ATC. **A** In glutamine rich status, cells use glutamine as a metabolite for cell growth. glutamine is recruited to tricarboxylic acid cycle, which controls antioxidant genes including GSH, and supply diverse nutrients to cells. **B** In glutamine metabolism inhibition via glutamine deprivation or BPTES treatment, antioxidant-related genes including GSH decrease leading to redox imbalance. Nonetheless, ATF4-dependent one-carbon metabolism makes redox imbalance mild and acts as an alternative strategy to tolerate to glutamine metabolism inhibition stress. **C** In co-inhibition of two pathways, excessive ROS aggravates the severity of anti-proliferation. it can increase lenvatinib and sorafenib-induced cell death through oxidative stress in ATC cells. The image of thyroid gland is acquired from MOTIFOLIO site.

To investigate the possible transition of one-carbon metabolism between ATC and PTC in detail, we performed single-cell RNA sequencing (scRNA-seq) analysis to compare levels of one-carbon metabolism genes in patients of ATC and PTC GEO datasets. During analysis total of 75,356 cells, we identified clusters of different cells including Thyrocyte with marker genes *TG*, *KRT18*; T cell with marker genes *CD3E*, *CD3G*; Fibroblast with marker genes *ACTA2*, *COL6A1*; Macrophage with marker genes *LYZ, IL1B*; Dendritic cell with marker genes *IRF4*, *IRF8*; B cell with marker genes *CD79A*, *CD19*; Endothelial cell with marker genes *CD93, CD34* (Fig. 7A, S9A). In integrated total of 24,728 thyrocyte cells, we next identified previously reported *CREB3L1*, *IGF2BP1*, EMT-related markers *SNAI2, TWIST1* upregulation and epithelial marker *CDH1* downregulation in ATC cluster (Fig. 7B) [45, 48]. Intriguingly, mRNA levels of total 11 one-carbon metabolism-related genes including upstream regulator *ATF4* were significantly higher than those in PTC patients (Fig. 7C). These findings led us to investigate the evolutionary transition of one-carbon metabolism from PTC to ATC progression in the RNA expression level using trajectory inference and pseudotime analysis. Beforehand, we observed PTC-related genes *LGALS3*, *NPC2* and *S100A13* were expressed along ATC cluster, suggesting that origin of ATC may be partially derived from PTC (Fig. S9B) [45]. We next performed trajectory inference and pseudotime analysis, appointing the top of ATC and PTC cluster area as root cells based on TDS score (Fig. 7D). Notably, our monocle3 tool analysis showed expression level of the average and several one-carbon metabolism-related genes increased along pseudotime from PTC to ATC (Fig. 7E, F and S9C, D). Expression level of these genes oppositely decreased according to TDS score, implying a possible increase of one-carbon metabolism according to evolutionary route from PTC to ATC progression (Fig. 7F and S9E–G). We also observed expression of PHGDH was higher in ATC compared to PTC patient tissues (Fig. 7G). Based on intensified one-carbon metabolism in ATC, we explored fate determination role of one-carbon metabolism in aggressive ATC and PDTC patients. We identified enrichment of poor prognosis-related genes in one-carbon metabolism high groups (Fig. 7H). High group of one-carbon metabolism genes had worse overall survival in ATC and PDTC patients (Fig. 7I). Additionally, we confirmed TDS score was negative correlation with the average of one-carbon metabolism genes and TDS score low group showed inferior survival, implying that one-carbon metabolism might be associated with poor prognosis in aggressive thyroid cancer (Fig. S10A, B). Collectively, our data suggest ATC might be evolved to employ one-carbon metabolism from PTC and this process is a potential regulator of tumorigenicity in aggressive thyroid cancer (Fig. 8).

## Discussion

Despite many candidate drugs continuously show chemotherapeutic possibilities, establishing proper targeted therapy remain unclarified on account of molecular drivers for tumorigenesis and high rate of metastasis in ATC patients [2, 49, 50]. Immunotherapy has recently arisen in the treatment of ATC such as spartalizumab and pembrolizumab targeting PD-1 and PD-L1 interaction. Nonetheless, limitations to immunotherapy showing discouraging results with chemotherapy and expensive cost lead to other strategy for overcoming ATC pathogenesis [51–53]. Therefore, investigating molecular details to improve in prognosis of ATC patients is indispensable.

ATC shows higher metabolic activity compared with other thyroid cancers. In addition, level of GLS1 and GDH is the highest in ATC among patients with thyroid cancer, we hypothesized inhibition of glutaminolysis might cause significant damage, leading to cell disruption in ATC cells [7, 54]. In publicly available CCLE and GEO datasets, we identified level of glutamine was significantly high among other cancer types and ATC was especially higher compared to non-ATC thyroid cell types [27]. Patients with ATC also had a higher expression of glutaminolysis-related genes than normal subjects or patients with PDTC.

However, we observed one-carbon metabolism serves as an endurable strategy to compensate for rapid turnover of 8505 C and SNU-80 under glutamine metabolism inhibition. There are several mechanisms by which cancer cells synthesize serine, a major donor of one-carbon metabolism under diverse environmental stresses. They tend to acquire serine via autophagy activation, glycolysis, uptake through transporters, and others [23, 26, 55, 56]. Therefore, it is necessary to investigate the main pathway driving serine synthesis under our experimental conditions. We also observed a decrease in the mRNA levels of *SHMT1* and *MTHFD1*, similar to previous papers. These proteins consume formate to entail several tumorigenic pathways in the cytosol. Therefore, the direct role of formate, a byproduct of one-carbon metabolism, on cell fate needs to be studied [55, 57, 58].

As cancer cells undergo metabolic reprogramming to adapt to nutrient changes, many investigations targeting cancer cell metabolism have been conducted [16, 59, 60]. Metabolic adaptation is usually achieved through changes in gene expression. This event occurs through epigenetic modification, which represents a rapid and reversible response and involves diverse enzymes including histone (de)methylases, (de)acetylases, and DNA. Indeed, genes on- and off-systems are rapidly controlled by dynamically changing chromatin architecture in response to nutrient stress [61, 62]. Our bioinformatic data analysis showed glutamine deprivation increased the transcription start site and RefSeq functional element peak, which suggests an elevation of transcription factor activity via chromatin opening. We confirmed ATF4 levels were notably increased, and therefore, one-carbon metabolism was positively controlled in the genetic inhibition experiment.

GSH is a predominant enzyme protecting cancer cells from oxidative stress [41, 42]. We observed inhibition of glutamine metabolism decreased total GSH contents. GSH synthesis in cancer cells occurs via many signaling pathways involving NADPH production such as pentose phosphate pathway (PPP), and one-carbon metabolism [39, 63]. Previous studies showed effect of glutamine deprivation on PPP deactivation in cancer and ROS increase during PPP inhibition in thyroid cancer [10, 64, 65]. We accordingly consider the possibility that PPP is deactivated and thereby GSH contents are decreased upon glutamine deprivation despite our observation that one-carbon metabolism is responsible for GSH loss. Based on predominant antioxidant function of GSH, we hypothesized GSH content loss is responsible for significant ROS accumulation in glutamine and one-carbon metabolism. We also demonstrated ROS is directly involved in cell proliferation following Trolox treatment. Our results were consistent with those of previously reported other cancer types such as glioblastoma or leukemia [22, 23].

Lenvatinib and sorafenib are chemotherapeutic reagents targeting multi-tyrosine kinases for ATC. Several clinical studies have reported monotherapy with a multi-tyrosine kinase inhibitor is not recommended for mild effects in patients with ATC [66, 67]. Recently, many investigations have shown combination treatment with these drugs is more effective than drug monotherapy for ATC [68–71]. We also showed co-inhibition of glutamine and one-carbon metabolism increased the efficacy of lenvatinib and sorafenib. In addition, combinational inhibition of glutamine and one-carbon metabolism synergistically induced ROS accumulation, which might contribute to tumor suppression with chemotherapy reagents. Despite the lack of reported cases in patients with ATC, glutamine levels in most cancers gradually decrease with increasing distance from a blood vessel, indicating the possible occurrence of one-carbon metabolism activation in the central tumor microenvironment [23, 72]. Thus, our concept of co-inhibition of glutamine and one-carbon metabolism may contribute to the development of a therapeutic strategy for ATC.

It was reported that PTC patients had a high expression of glycine and serine metabolism-related proteins such as PHGDH, PSAT1, PSPH and SHMT1 [73, 74]. In our analysis to study the one-carbon metabolism process between ATC and PTC, we observed one-carbon metabolism-related genes and serine family amino acid process in ATC were higher compared with PTC, which led us to hypothesize evolutionary transition might elicit enhancement of one-carbon metabolism in ATC. Indeed, single-cell analysis showed one-carbon metabolism-related genes in ATC cluster were higher more than PTC. Furthermore, pseudotime inference analysis based on TDS score exhibited the increase of average and several one-carbon metabolism genes along the pseudotime from PTC to ATC. In ATC and PDTC groups with inferior prognosis compared with well-differentiated PTC, we confirmed one-carbon metabolism might contribute to poor prognosis in aggressive thyroid cancer. In conclusion, our study represent ATC might increase the availability of one-carbon metabolism leading to escape from diverse stresses such as metabolic stress and anticancer drug.

## Materials and methods

### Antibodies and reagents

BPTES (HY-12683), CBR-5884 (HY-100012), SHIN1 (HY-112066), DS18561882 (HY-130251), and sorafenib (HY-10201) were purchased from MedChemExpress. Trolox (S3665), lenvatinib (S1164) were from selleckchem. Crystal violet (V5265), H_2_DCFDA (D399), MitoSOX^TM^ Red (M36008), monobromobimane (mBBr, M1378), Propidium Iodide (P3566), RNase A (10109169001), (3-(4,5-Dimethylthiazol-2-yl)-2,5-Diphenyltetrazolium Bromide) (MTT, M6494), Hoechst 33342 (H3570), and Lipofectamine^TM^ RNAiMAX Transfection Reagent (13778150) were obtained from Thermo Fisher Scientific. H_2_O_2_ (216763) was purchased from Sigma-Aldrich. Triton X-100 (0694) was purchased from AMRESCO. Primary antibodies against SHMT2 (sc-390641), PHGDH (sc-100317), and β-actin (sc-47778) were purchased from Santa Cruz Biotechnology. MTHFD2 (98116 S) was acquired from Cell Signaling Technology. Secondary antibodies of the mouse and rabbit were from Cell Signaling Technology and Abcam respectively (7076 S and ab6721). Small interfering RNA (siCTRL and siATF4) oligonucleotides were purchased from Santa Cruz Biotechnology (sc-37007 and sc-35112, respectively).

### Cell culture and treatment

8505 C cells obtained from the European Collection of Authenticated Cell Cultures (ECACC) were grown in DMEM medium (Corning, 10-013-CV)-1% penicillin-streptomycin (Gibco, 15140122) supplemented with 10% fetal bovine serum (Corning, 35-015-CV). SNU-80 cells acquired from Korean Cell Line Bank (KCLB) were grown in RPMI medium (Corning, 10-041-CV)-1% penicillin-streptomycin (Gibco, 15140122) supplemented with 10% fetal bovine serum (Corning, 35-015-CV). The cells were tested for mycoplasma elimination using a Plasmocin solution (Invivogen, ant-mpt). For in vitro treatment, following concentrations were employed: Glutamine-full medium consists of DMEM or RPMI medium (Welgene, LM-001-05 or Corning, 10-041-CV)-1% penicillin-streptomycin (Gibco, 15140122) supplemented with 10% fetal bovine serum (Corning, 35-015-CV); Glutamine-free medium consists of DMEM or RPMI medium (Welgene, LM-001-08 or LM-011-05)-1% penicillin-streptomycin (Gibco, 15140122) supplemented with 10% dialyzed fetal bovine serum (Gibco, 26400044); MTT, 0.2 (8505 C) or 0.5 (SNU-80) mg/ml; Crystal violet solution, 0.1%; Hoechst 33342, 2 ug/ml; mBBr, 10 μM; H_2_DCFDA, 10 μM; MitoSOX^TM^ Red, 5 μM; BPTES, 10 (8505 C) or 5 (SNU-80) μM; CBR-5884, 60 μM; Trolox, 25 μM; lenvatinib, 50 (8505 C) or 25 (SNU-80) μM; sorafenib, 10 μM; H_2_O_2_, 10 μM.

### Cell proliferation and viability assay

For cell proliferation assay, 8505 C and SNU-80 cells (5 × 10^3^ and 3 × 10^3^ cells/well) seeded in 96-well plates were exposed to glutamine-free medium or reagents for the indicated times, with the vehicle as a control. For cell viability assay, 8505 C and SNU-80 cells (1 × 10^4^ and 5 × 10^3^ cells/well) grown in 96-well plates were treated with glutamine-free medium or reagents for the indicated times. Cells were incubated for 2 h in a 37 °C incubator after the addition of 10 µl of MTT solution and formazan was dissolved in 50 µl DMSO (Sigma, 34943). The absorbance was measured at 570 nm using a Multiskan GO spectrophotometer (Thermo Fisher Scientific, 51119300).

### Colony formation assay

8505 C cells (5 × 10^3^ cells/well) were seeded in 6-well plates and treated with glutamine-free medium, BPTES, for 10 days. After washing with PBS twice, cells were fixed with cold methanol at room temperature for 5 min and stained with crystal violet for 15 min. After the crystal violet was slowly removed, the plates were air-dried overnight. Each sample was then added to 1 ml of methanol and rotated for 20 min. The optical density of each well was measured at 570 nm using a Multiskan GO spectrophotometer (Thermo Fisher Scientific, 51119300).

### LDH assay

8505 C and SNU-80 cells (5 × 10^3^ and 3 × 10^3^ cells/well) were seeded in a 96-well plate. After 24 h, the cells were treated with glutamine-free medium or BPTES for the indicated times. Cell death was assessed based on the release of LDH into the extracellular medium, which was measured using a Cytotoxicity Detection Kit according to the manufacturer’s protocol (Thermo Fisher Scientific, C20301).

### Analysis of nuclear fragmentation by Hoechst 33342 staining

8505 C cells (1 × 10^5^ cells/well) grown on a chambered coverglass (Thermo Fisher Scientific, 154526) were treated with glutamine-free medium, BPTES, and H_2_O_2_ for 24 h. After staining with Hoechst 33342 solution for 10 min in a 37 °C incubator, cells were washed with PBS and fixed with 4% paraformaldehyde at room temperature for 10 min. Fragmented cells were measured using fluorescence microscopy (BX53F, OLYMPUS, Japan).

### Sample preparation for LC-MS metabolomic analysis

8505 C cells (8 × 10^5^ cells/well) were seeded in 100 mm cell culture dish and treated with glutamine-free medium or BPTES for 72 h. After aspirating cell medium, cells were washed with 10 ml pre-cool PBS twice on ice. Next, pre-cool PBS was removed and 1 ml pre-chilled 80% methanol was added immediately. After scraping cells with a cell scraper on ice, mixture of cell lysate and methanol was transferred to 1.5 ml tubes and stored at −80 °C deep freezer for overnight.

### Liquid chromatography

UPLC separation was performed on a Thermo Scientific™ UltiMate 3000 RSLC system using Acquity UPLC BEH C18, 1.7 μm, 2.1 × 100 mm. The flow rate and operating temperature are 300 μl/min and 50 °C respectively. The LC solvent of phase A is 0.1% formic acid in distilled water and phase B is 0.1% formic acid in acetonitrile. Linear gradient was implemented from 5% B for 2 min, followed by increasing to 100% at 8 min, hold 100% B for 4 min, then decreasing to 5% at 0.5 min, then equilibrate for another 2.5 min. The sample injection volume is 5 μl.

### Mass spectrometry

The Thermo Scientific™ Q Exactive Orbitrap Plus™ mass spectrometer was operated under electrospray ionization (ESI) positive mode. Full scan type (80–1000 m/z) used resolution 70,000. Data-dependent MS/MS were acquired on a “Top5” data-dependent mode using the following parameters: resolution 17,500; 2 amu isolation window; normalized collision energy 30; dynamic exclusion 6 s. Source ionization parameters were: spray voltage, 3.5 kV; capillary temperature, 370 °C; and S-Lens level, 55.

### Real-time RT-PCR

RNA of 8505 C cells was isolated using TRIzol reagent (Invitrogen, 15596018). cDNA was synthesized using 1 ug total RNA and ImProm-II™ Reverse Transcriptase (Promega, A3803). Real-time RT-PCR was performed using TOP Real ™ qPCR 2X Pre-MIX (Enzynomics, RT501S) and specific primers in a CFX Connect Real-Time PCR instrument (Bio-Rad, 1855201). Gene expression was normalized to the *36B4* mRNA expression levels.

The primer sequences for real-time RT-PCR are listed in Supplementary Table 1.

### Immunoblot assays

Protein lysates were lysed in mammalian lysis buffer (25 mM Tris-HCl, pH 7.8, 150 mM NaCl, 0.1% NP-40, 1 mM EDTA, 10% glycerol) supplemented with Xpert Protease Inhibitor Cocktail Solution (GenDEPOT, P3100-001). Protein concentrations were quantified using the Bio-Rad Protein Assay Kit (#5000006). Samples were separated by SDS-PAGE (8-10%) and transferred onto a nitrocellulose membrane (Amersham, 10600001). Membranes blocked with 5% skim milk-Tris-0.1% Tween 20 for 30 min and incubated diluted antibodies at 4 °C overnight. After incubation with HRP-conjugated secondary antibodies for 1 h at room temperature, immunoblot signals were detected using Clarity Western ECL Substrate (BR1705061 and 1705062). For gene silencing with siCTRL or siATF4, the cells were transfected for 48 h with Lipofectamine^TM^ RNAiMAX Transfection Reagent according to the manufacturer’s protocol.

### Study design and ethical considerations

This study was a retroactive, single center examination of patients diagnosed with ATC (September 2021–January 2022). All courses entailing patients were achieved in proportion to the institutional ethical standards, whole applicable national/local regulations, and guidelines of the 1964 Helsinki Declaration and its later amendments. The study procedures were authorized by the Institutional Review Board (IRB) of Gangnam Severance Hospital, Yonsei University College of Medicine (IRB protocol: 3-2021-0043).

### IHC staining

After the samples were deparaffinized and rehydrated, sections for PHGDH staining were placed in FLEX Target solution (DAKO, K8004 (pH9.0)) for antigen retrieval by boiling in a PT link for 20 min at 95 °C. For inactivation of endogenous peroxidase, sections were treated with 3% H_2_O_2_ for 10 min and washed with TBS for 5 min twice. Next, the slides were incubated with PHGDH antibody (1:100) for 1 h at room temperature. After washing three times with TBS for 5 min each, the slides were incubated with the secondary antibody (DAKO, K4003) for 20 min at room temperature. Diaminobenzidine (DAB) (DAKO, K3468) was used for color development for 5 min. Finally, the slides were counterstained with hematoxylin for 10 min, dehydrated, and mounted. Quantitative data for comparing PHGDH expression in patients were analyzed using Qupath software. (version 0.4.2).

### Measurement of ROS and GSH contents

To measure intracellular ROS, 8505 C (2 × 10^5^ cells/well) and SNU-80 cells (8 × 10^4^ cells/well) were seeded in a 12-well plate. After 24 h, the cells were treated with the indicated drugs; vehicle (DMSO), BPTES, CBR-5884, lenvatinib and sorafenib for the indicated times. Then, the cells were stained with H_2_DCFDA for 30 min at 37 °C incubator. For mitochondrial ROS measurement, 8505 C cells were stained with MitoSOX^TM^ Red for 30 min at 37 °C incubator. To determine the total GSH content, 8505 C cells were stained with mBBr for 10 min in a 37 °C incubator. Fluorescence intensity was quantified by flow cytometry (FACSVerse, BD Biosciences) within 30 min, and the data were analyzed using FlowJo software (version 10.4.2).

### Cell cycle assay

8505 C cells (1 × 10^5^ cells/well) seeded in 12-well plates were treated with glutamine-free medium and BPTES for 24 h. Then, cells were harvested and fixed with 70% ethanol at 4 °C overnight. After washing with PBS, cells were incubated with PBS containing 20 ug/ml of Propidium Iodide, 0.2 mg/ml of RNase A, 0.1% Triton X-100 for 15 min at 37 °C incubator. Cell cycle progression was quantified by flow cytometry (FACSVerse, BD Biosciences), and the data analysis was performed using FlowJo software (version 10.4.2).

### RNA sequencing

Total RNA samples of 8505 C cells were duplicated, and processing was performed by Macrogen Inc. (Seoul, Korea; www.macrogen.com). First, a library was constructed using the TruSeq Stranded mRNA LT Sample Prep Kit (Illumina, San Diego, CA, USA) according to the TruSeq Stranded mRNA Sample Preparation Guide (part #15031047 Rev. E). Next, sequencing was performed following the NovaSeq 6000 System User Guide (Document #1000000019358 v02). The sequence was qualified by FastQC (version 0.11.7), trimmed by Trimmomatic (0.38), and mapped using the HISAT2 (version 2.1.0) program. We assembled gene and transcript expression levels to read counts or fragments per kilobase of transcript per million mapped reads (FPKM) using StringTie (version 2.1.3b). Trimmed mean of M-value (TMM) normalization was performed to reduce systematic bias using read count by the edgeR package library. Finally, the DEGs were estimated using edgeR.

### ATAC sequencing

Total RNA samples of 8505 C cells were duplicated, and processing was performed by Macrogen Inc. (Seoul, Korea; www.macrogen.com). The sequence was qualified using FastQC (version 0.11.7), trimmed using Trim Galore (version 0.5.0), and aligned using the Bowtie2 (version 2.3.5.1) tool. Peak calling from alignment bam files was performed using MACS2 (version 2.1.1.20160309). Raw BAM files were normalized using the Galaxy tool based on the hg38.blacklist.bed file. The total peak signals from the raw bed files are depicted by ShinyCircos. Transcription start site or Functional elements peak signals were analyzed from computeMatrix in Galaxy tool. These two open sources were acquired from UCSC Genome Browser. The peak signals for each gene were visualized using GBiB tool.

### Acquisition of scRNA-seq data

We obtained all the findings of single-cell analysis from the GEO database. The scRNA-seq data consisting of thyrocytes from ATC (*n* = 5) and PTC patients (*n* = 7) were acquired from GSE148673 and GSE184362 datasets respectively. ‘Seurat (version 4.3.0)’ and ‘DoubletFinder (version 2.0.3)’ packages in R software (version 4.1.3) were used for scRNA-seq data preprocessing. First, scRNA-seq expression matrices were inserted into R using ‘Read10X’. Quality control of each data was performed to filter out poor-quality cells based on numbers of genes per cell (nFeature), whole number of read counts (nCount) and the read counts percentage of mitochondrial genes per cell (percent_MT). After eliminating doublets using ‘doubletFinder_v3’, the scRNA-seq data were normalized by the ‘LogNormalize’, and the top 2000 highly variable genes were identified using ‘FindVariableFeatures’. Integration of each data was performed using ‘IntegrateData’. Information about quality control and percent of doublet are represented in Supplementary Table 2 in more detail.

### Processing of the scRNA-seq data

Cell cycle score of each single cell was calculated using ‘CellCycleScoring’ and integrated data were regressed out to mitigate cell cycle heterogeneity on cell clustering. PCA was utilized with the top fifteen PCA values for dividing clusters using ‘RunPCA’. Total seven major cell types were annotated to each cluster according to marker gene expression through ‘FindAllMarkers’. Cluster visualization was performed using UMAP algorithm (resolution=0.15). ‘DotPlot’ was used to plot marker genes to identify each cell cluster identity. ‘RunALRA’ were performed to recover missing values in cluster of thyrocytes. ‘FeaturePlot’ was used to compare several genes related to cancer type-specific markers and one-carbon metabolism between ATC and PTC.

### Trajectory inference and pseudotime analysis based on TDS score

To distinguish ATC and PTC in Thyrocyte Cluster, we first calculated TDS score using ‘AddModuleScore’ and further investigated expression level of several genes. Next, we assigned starting point of the pseudotime analysis based on TDS score using Monocle3 (version 1.3.1) package. Scatterplot of one-carbon metabolism genes was created using ‘FeatureScatter’ along TDS score and pseudotime from PTC to ATC.

### Statistical analysis

Data are expressed as the mean ± SD of three independent experiments. A two-tailed Student’s *t* test was used to compare the values between the two groups. One-way ANOVA with Tukey’s test was used to compare multiple groups. Statistical significance was set at *P* < 0.05. Data were analyzed using the GraphPad Prism 9 software. The mathematical formula for drug synergy calculation was acquired from the SynergyFinder (https://synergyfinder.fimm.fi/synergy/synfin_docs/) with HSA and Bliss model.

## Supplementary information

Supplementary figures and tables

Original data files

Reproducibility Checklist

## Data Availability

The raw sequencing data of bulk RNA Seq and ATAC-Seq generated in this study are available under NCBI Sequence Read Archive (SRA). The SRA code is PRJNA954580. The data acquired or analyzed during the current study and materials are available from the corresponding author without imposing restrictions on reasonable requests.
