# Supplementary material for: Co-inhibition of glutaminolysis and one-carbon metabolism promotes ROS accumulation leading to enhancement of chemotherapeutic efficacy in anaplastic thyroid cancer
Source: Cell Death Dis. 2023 Aug 12;14(8):515. doi: 10.1038/s41419-023-06041-2 (PMC10423221; doi:10.1038/s41419-023-06041-2)
Supplement: Supplementary file 1 — Supplementary figures and tables [file 41419_2023_6041_MOESM1_ESM.docx]

**
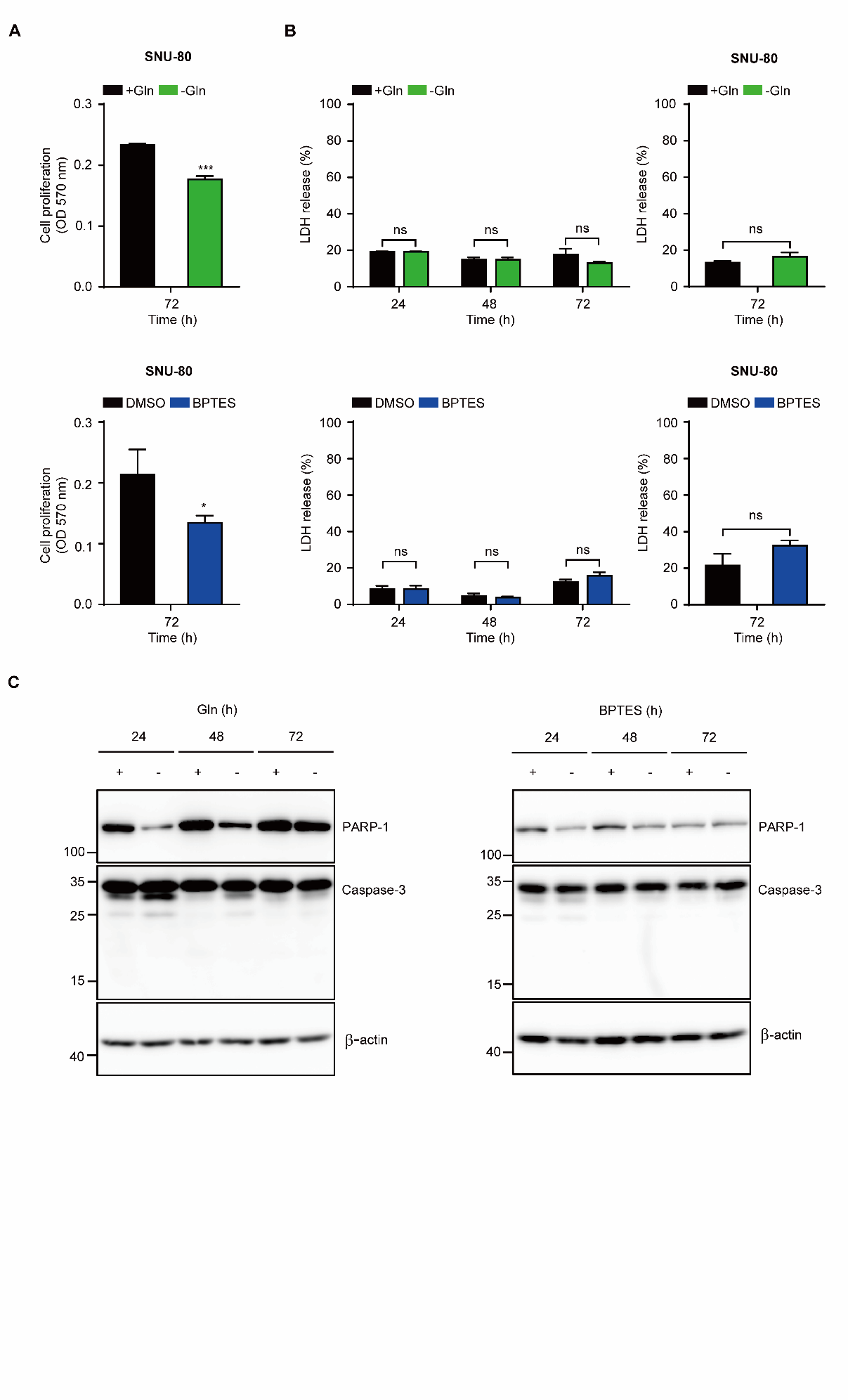
**

**Supplementary Fig. 1 Glutamine metabolism inhibition fails to induce cell death in ATC cells. A** SNU-80 cells were exposed to glutamine deprivation medium or BPTES (5 uM) for 72 h. Cell proliferation was measured by MTT. **B** Cells were treated with glutamine-free medium or BPTES (8505C; 10 uM) or (SNU-80; 5 uM) for 72 h. Cytotoxicity was measured by LDH assay. **C** Western blots show PARP-1, Caspase-3, β-actin treated with glutamine deprivation or BPTES for 72 h. The number left to the immunoblot images indicates the protein size of the immunoband measured for this analysis. Data are expressed as the mean S.D. of three independent experiments (n=3). Statistical comparisons were performed using two-tailed Student’s t-test. (ns, not significant) (Full and uncropped western blots can be found in original data files).


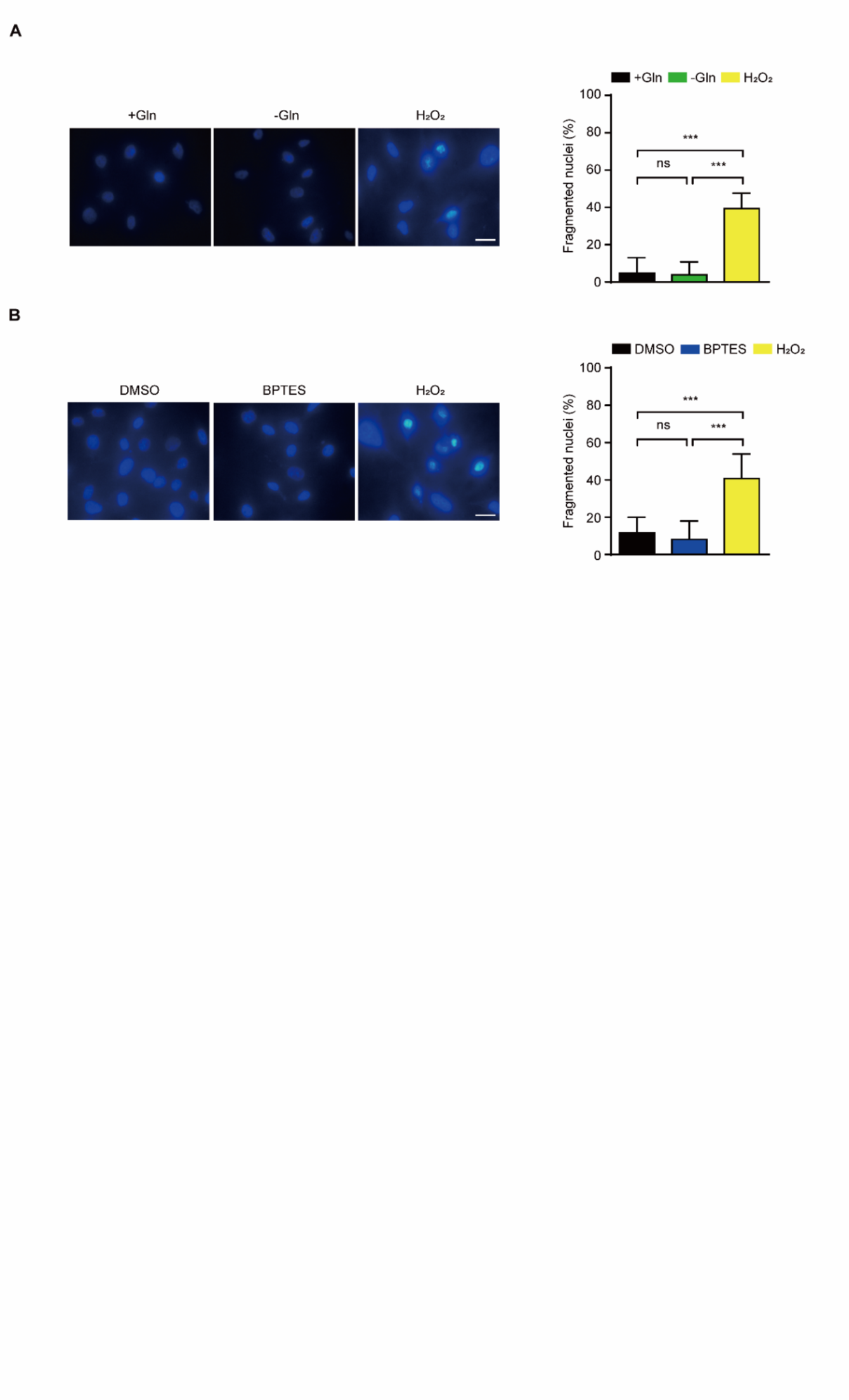


**Supplementary Fig. 2 Glutamine metabolism inhibition does not trigger DNA fragmentation in 8505C. A, B** 8505C cells were treated with glutamine deprivation or BPTES (10 uM) for 24 h respectively. H_2_O_2_ (10 uM) was used as a positive control. Randomly pictured numbers for quantifying percentage of fragmented nuclei is 10. Scale bar = 20 um. Data are expressed as the mean S.D. of three independent experiments (n=3). Statistical comparisons were performed using ANOVA followed by Tukey’s multiple comparison test. (****P* < 0.001; ns, not significant)­­­


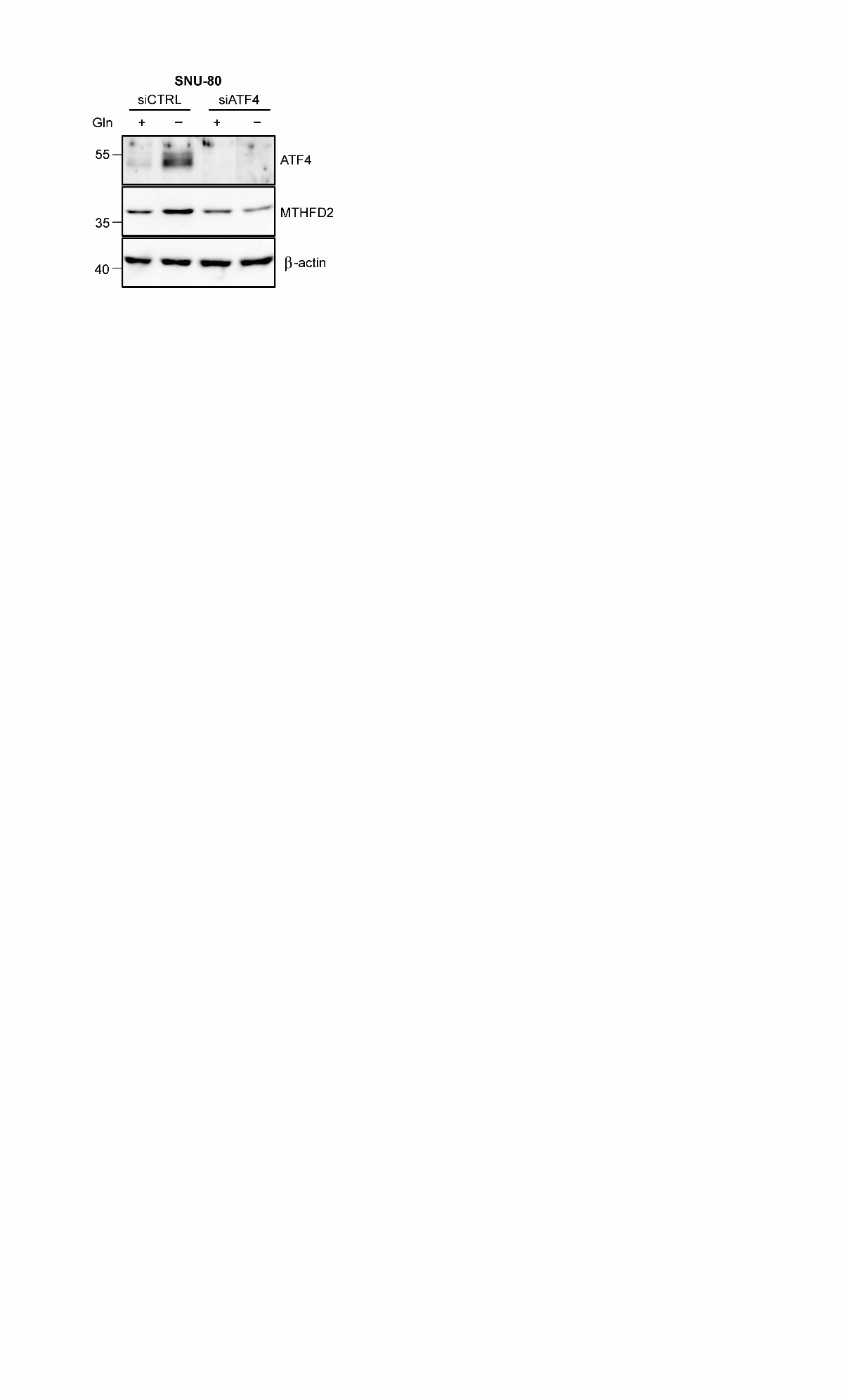


**Supplementary Fig. 3 Immunoblot assay of ATF4, MTHFD2, and β-actin in siCTRL and siATF4-transfected cells under glutamine deprivation for 24 h in SNU-80 cells.** The number left to the immunoblot images indicates the protein size of the immunoband measured for this analysis. (Full and uncropped western blots can be found in original data files).


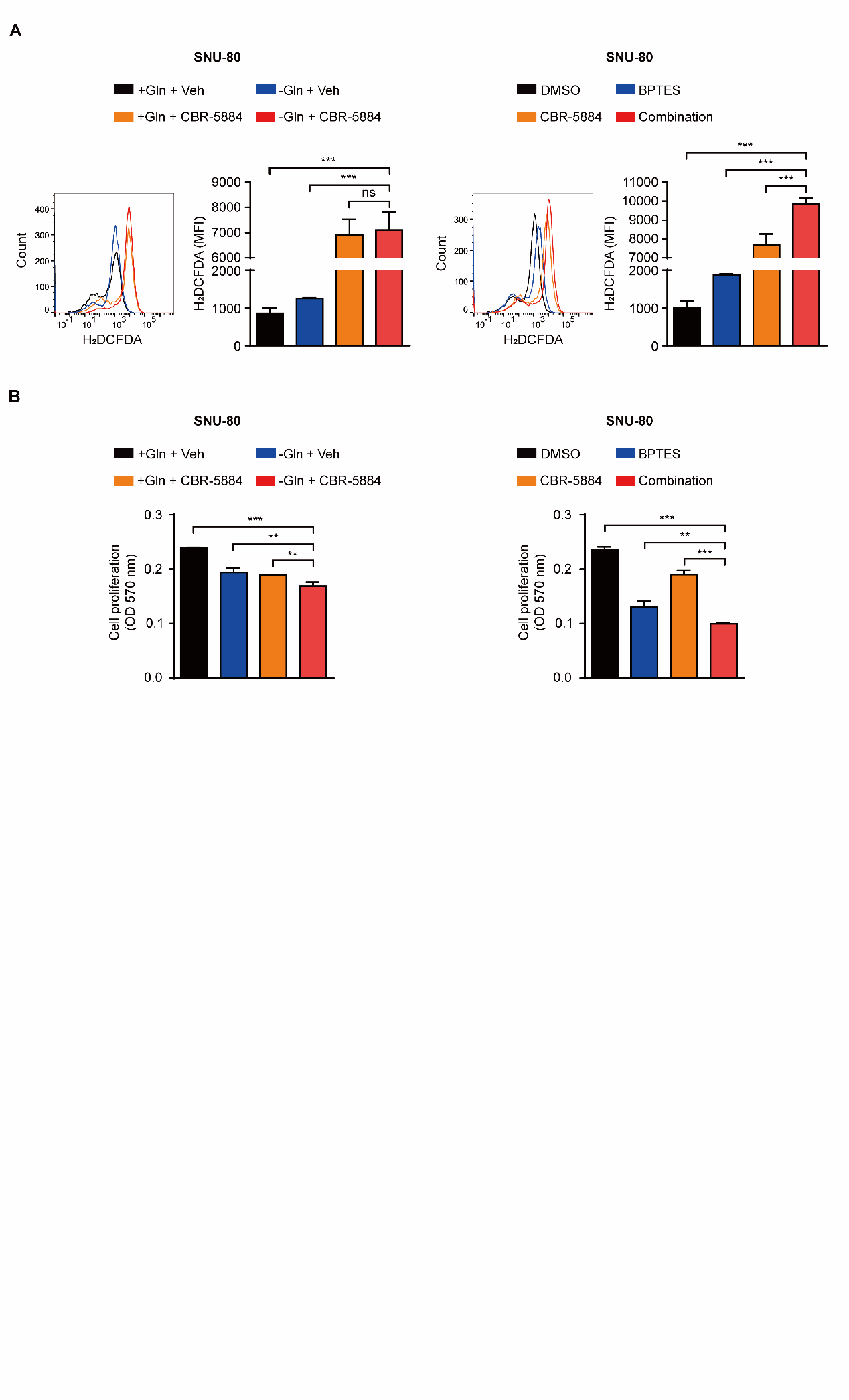


**Supplementary Fig. 4 Co-inhibition of glutamine and one carbon metabolism increases ROS level and reduces cell proliferation the most in SNU-80. A** Cells were treated with indicated groups (glutamine-free medium, BPTES; 5 uM, CBR-5884; 60 uM) for 24 h. Bar graph represents intracellular ROS level in indicated groups by H_2_DCFDA staining (10 uM) in flow cytometry. Representative histograms are shown (left panel). **B** Cells were treated with indicated groups (glutamine-free medium, BPTES; 5 uM, CBR-5884; 60 uM) for 72 h. Cell proliferation was measured by MTT. Data are expressed as the mean S.D. of three independent experiments (n=3). Statistical comparisons were performed using ANOVA followed by Tukey’s multiple comparison test. (***P* < 0.01; ****P* < 0.001; ns, not significant)­­­

**
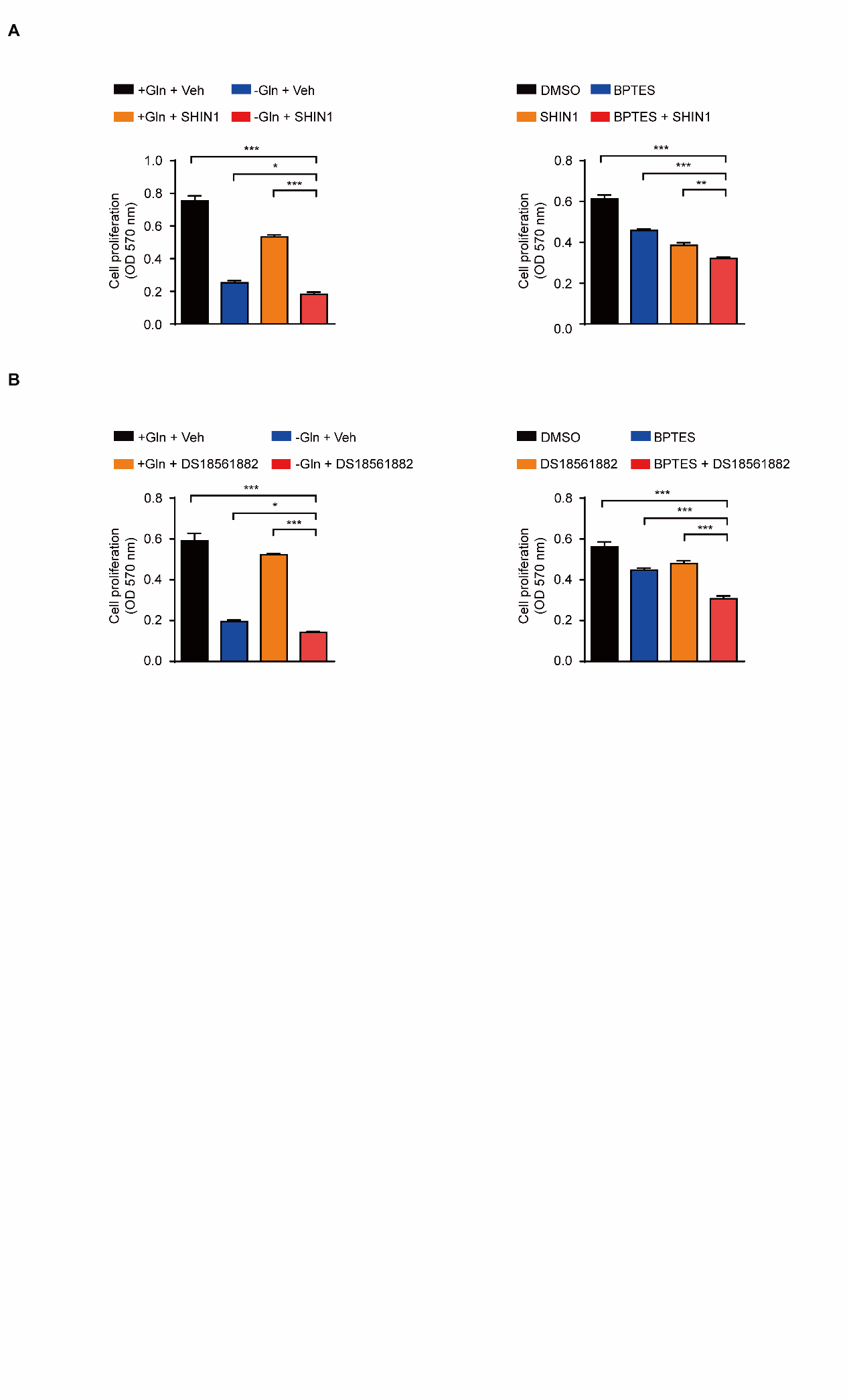
**

**Supplementary Fig. 5 Other one carbon metabolism inhibitor SHIN1 and DS18561882 promote cell proliferation arrest under glutamine metabolism inhibition in 8505C. A** Cells were treated with SHIN1 under glutamine metabolism inhibition for 72 h. Cell proliferation was measured by MTT. **B** Cells were treated with DS18561882 under glutamine metabolism inhibition for 72 h. Cell proliferation was measured by MTT. Data are expressed as the mean S.D. of three independent experiments (n=3). Statistical comparisons were performed using ANOVA followed by Tukey’s multiple comparison test. (**P* < 0.05; ***P* < 0.01; ****P* < 0.001)­­­


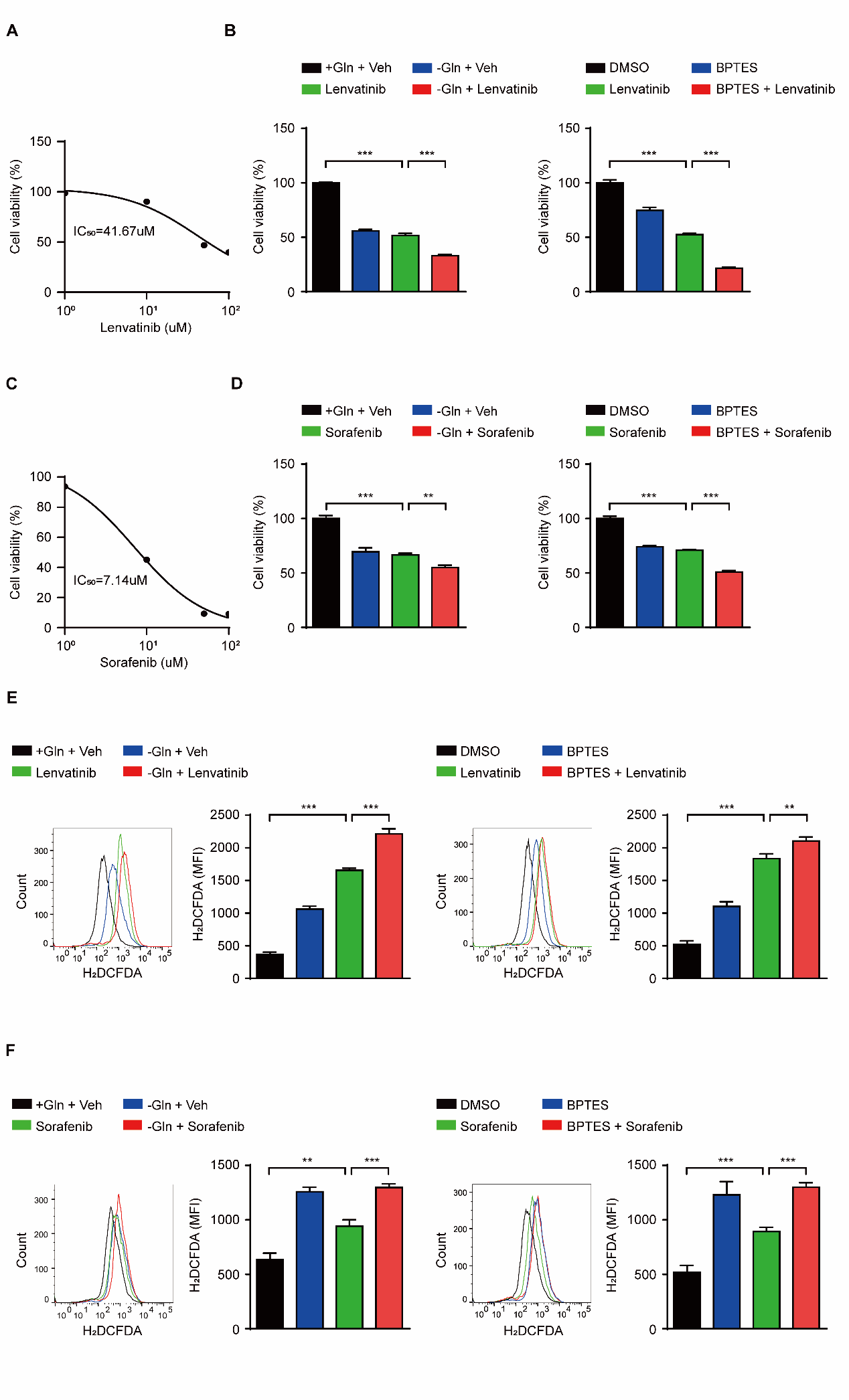


**Supplementary Fig. 6 Glutamine metabolism inhibition alone increases chemotherapy efficacy and drug-induced ROS accumulation in 8505C.** **A** Cells were treated with lenvatinib in a dose-range 1-100 uM for 48 h. IC_50_ value of lenvatinib was calculated using GraphPad Prism 9 software. **B** Cells were treated with indicated groups (glutamine-free medium, lenvatinib; 50 uM; BPTES; 10 uM) for 48 h. Cell viability was measured by MTT. **C** Cells were treated with sorafenib in a dose-range 1-100 uM for 48 h. IC_50_ value of sorafenib was calculated using GraphPad Prism 9 software. **D** Cells were treated with indicated groups (glutamine-free medium, sorafenib; 10 uM, BPTES; 10 uM) for 48 h. Cell viability was measured by MTT. **E** Cells were treated with indicated groups (glutamine- free medium, lenvatinib; 50 uM, BPTES; 10 uM) for 9 h. Bar graph shows intracellular ROS level in indicated groups by H_2_DCFDA staining (10 uM) in flow cytometry. Representative histograms are shown (left panel). **F** Cells were treated with indicated groups (glutamine-free medium, Sorafenib; 10 uM, BPTES; 10 uM) for 9 h. Bar graph shows intracellular ROS level in indicated groups by H_2_DCFDA staining (10 uM) in flow cytometry. Representative histograms are represented (left panel). Data are expressed as the mean S.D. of three independent experiments (n=3). Statistical comparisons were performed using ANOVA followed by Tukey’s multiple comparison test. (***P* < 0.01; ****P* < 0.001)


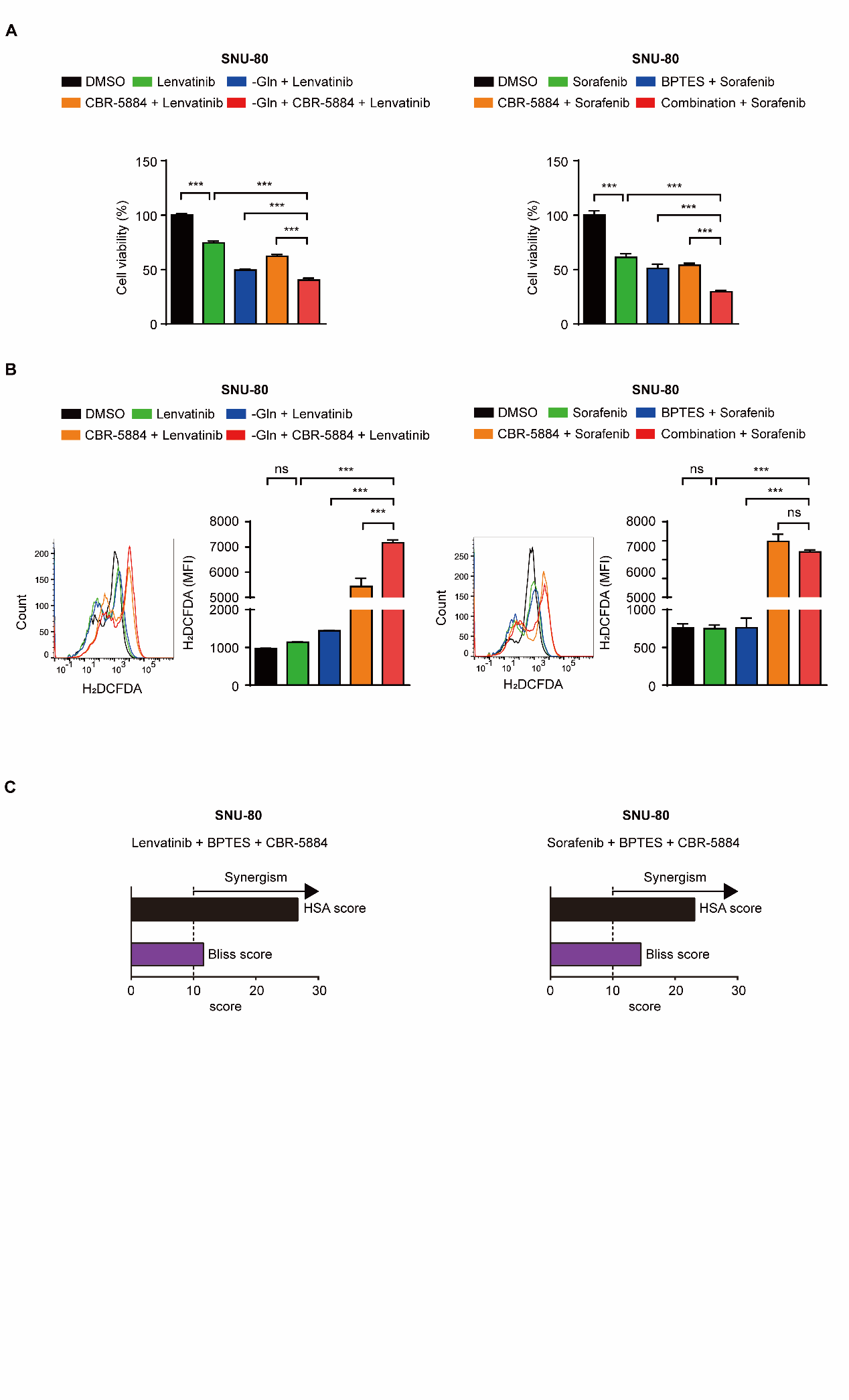


**Supplementary Fig. 7 Combined targeting of glutaminolysis and one carbon metabolism synergistically enhances chemotherapy efficacy by ROS overload in SNU-80.** **A** Cells were treated with indicated groups (lenvatinib; 25 uM; sorafenib; 10 uM; BPTES; 5 uM, CBR-5884; 60 uM) for 48 h. Cell viability was measured by MTT. **B** Cells were treated with indicated groups (lenvatinib; 25 uM; sorafenib; 10 uM; BPTES; 5 uM, CBR-5884; 60 uM) for 16 (lenvatinib) or 9 (sorafenib) h. Bar graphs exhibit intracellular ROS level in indicated groups by H_2_DCFDA staining (10 uM) in flow cytometry. Representative histograms are shown (left panel). **C** Cells were treated with indicated single drug (lenvatinib; 25 uM; sorafenib; 10 uM; BPTES; 5 uM; CBR-5884; 60 uM) for 48 h. The synergistic effects of multiple drugs were calculated using HSA and Bliss model. Data are expressed as the mean S.D. of three independent experiments (n=3). Statistical comparisons were performed using ANOVA followed by Tukey’s multiple comparison test. (****P* < 0.001; ns, not significant)

**
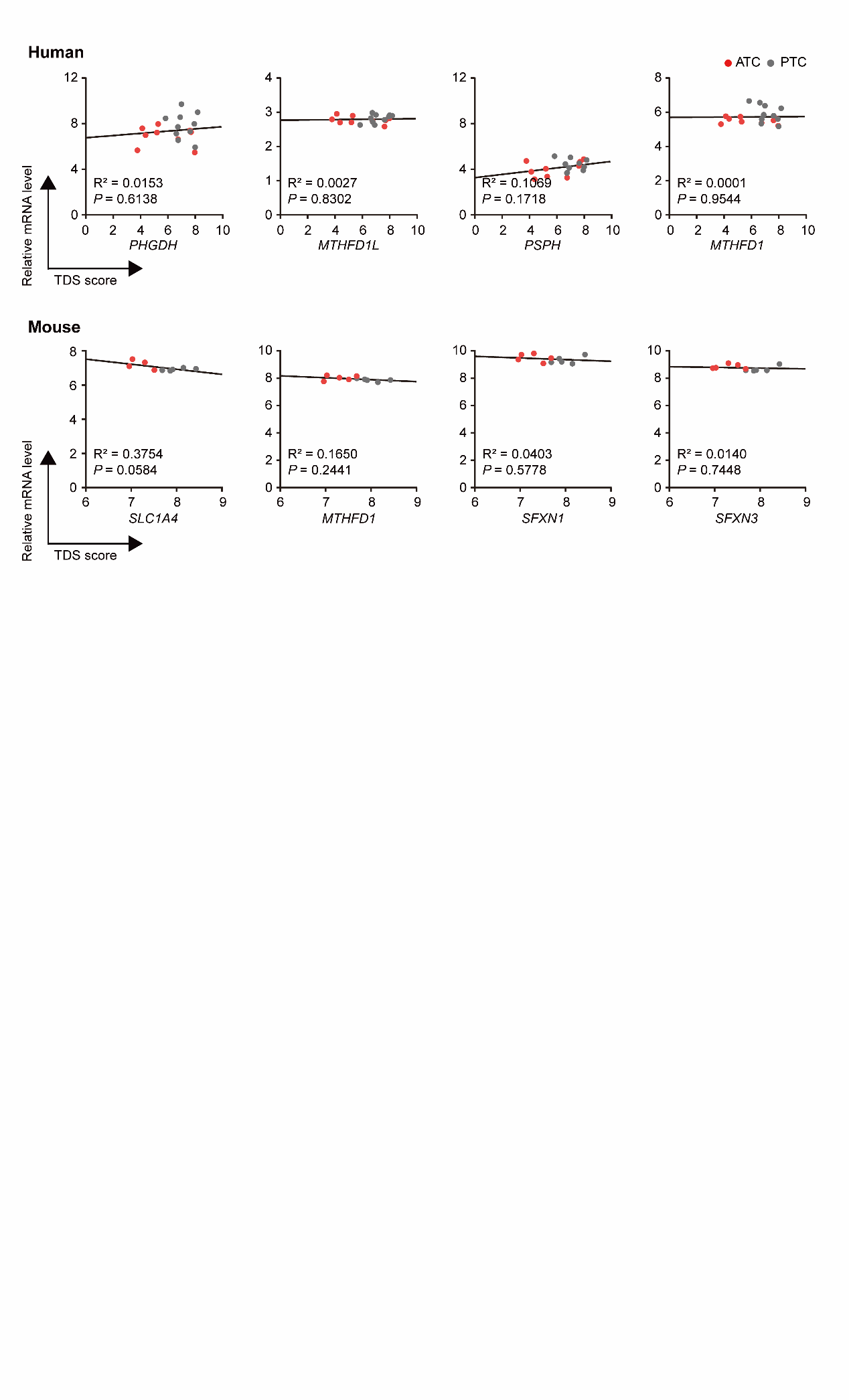
**

**Supplementary Fig. 8 Several one carbon metabolism genes of ATC and PTC show no correlation with TDS score in human and mouse datasets.** The black lines indicate simple linear regression. (*x*-axis) : TDS score; (*y*-axis) : relative mRNA level of several one carbon metabolism genes.

**
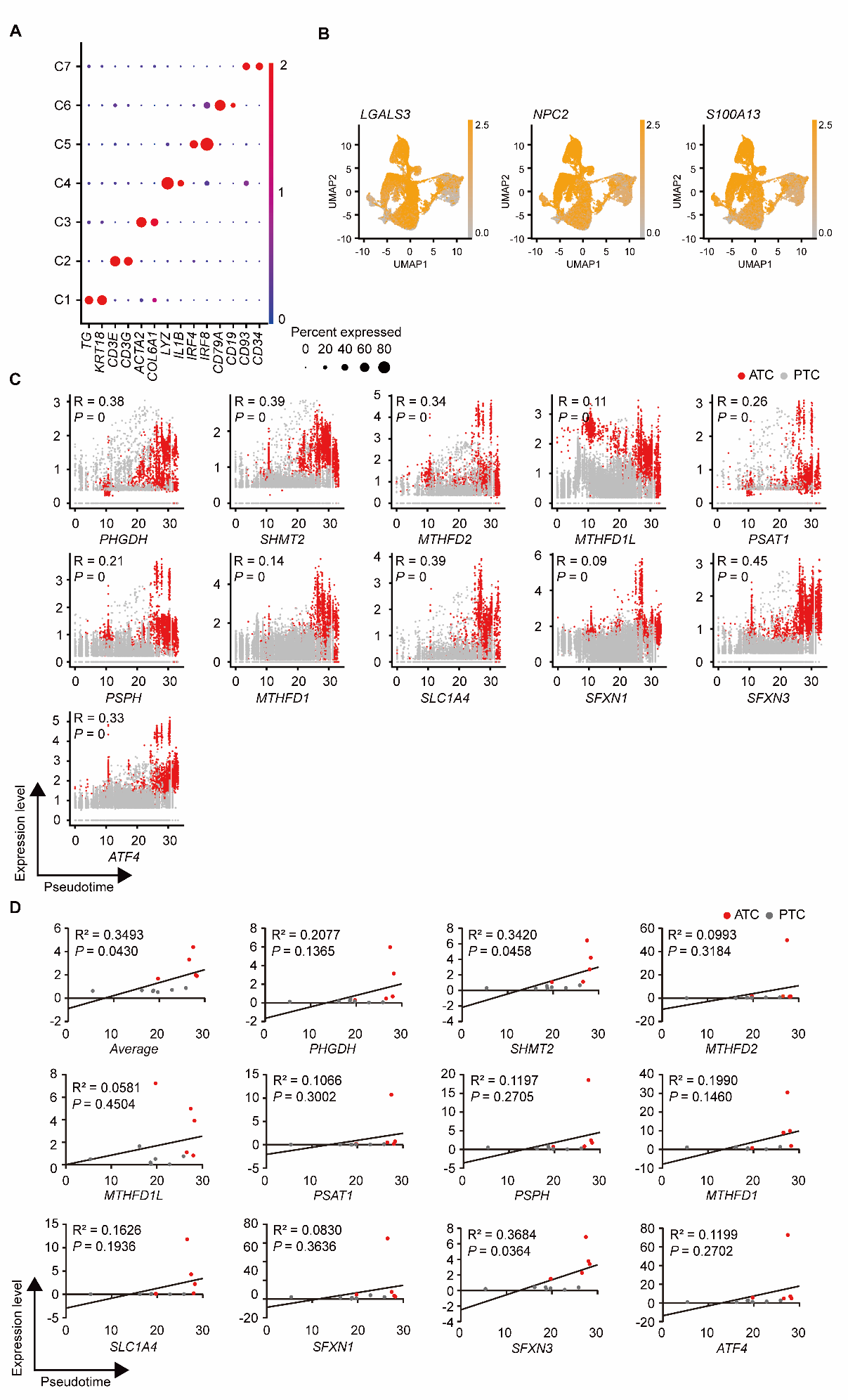
**

**­
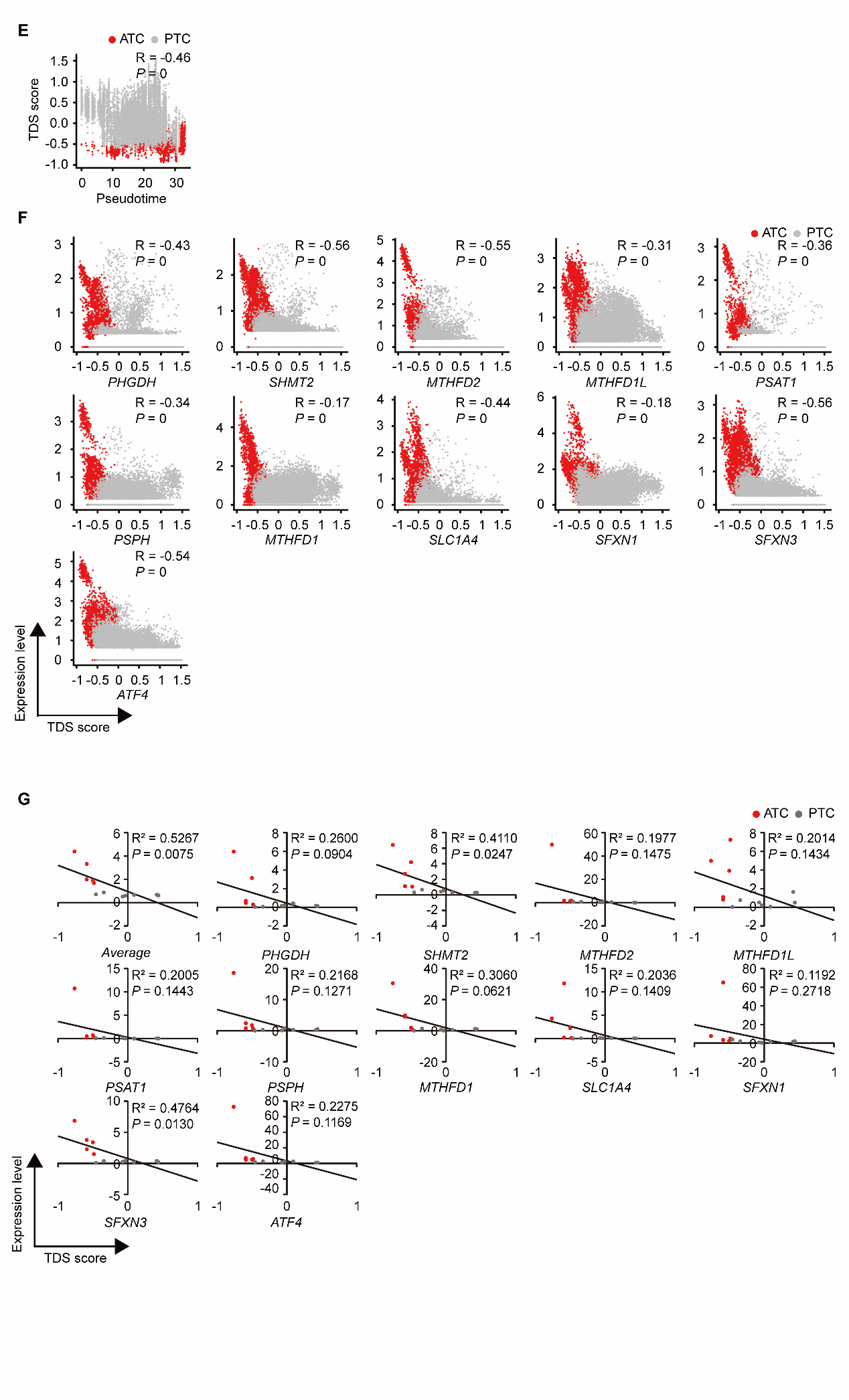
**

**Supplementary Fig. 9 Characteristics of cellular state in single cell analysis. A** Dot plot shows dot size representing the marker expression percentage of cells and the color scale means the average marker expression in the ATC and PTC total clusters. **B** UMAP show the levels of PTC-related genes *LGALS3, NPC2, S100A13*. The orange color scale represents expression level of each gene. **C** Scatterplot represents positive correlation between expression level of total eleven one carbon metabolism genes (*y*-axis) and pseudotime (*x*-axis) in patients with ATC and PTC. **D** Graphs show the correlation between expression level of one carbon metabolism genes (*y*-axis) and pseudotime (*x*-axis) in patients with ATC and PTC. Each dot indicates the mean value of each patient. The black lines indicate simple linear regression. **E** Scatterplot exhibits negative correlation between TDS score (*y*-axis) and pseudotime (*x*-axis) of ATC and PTC patients. **F** Scatterplot exhibits negative correlation between expression level of total eleven one carbon metabolism genes (*y*-axis) and TDS score (*x*-axis) in patients with ATC and PTC. **G** Graphs show the correlation between expression level of one carbon metabolism genes (*y*-axis) and TDS score (*x*-axis). Each dot indicates the mean value of each patient. The black lines indicate simple linear regression.


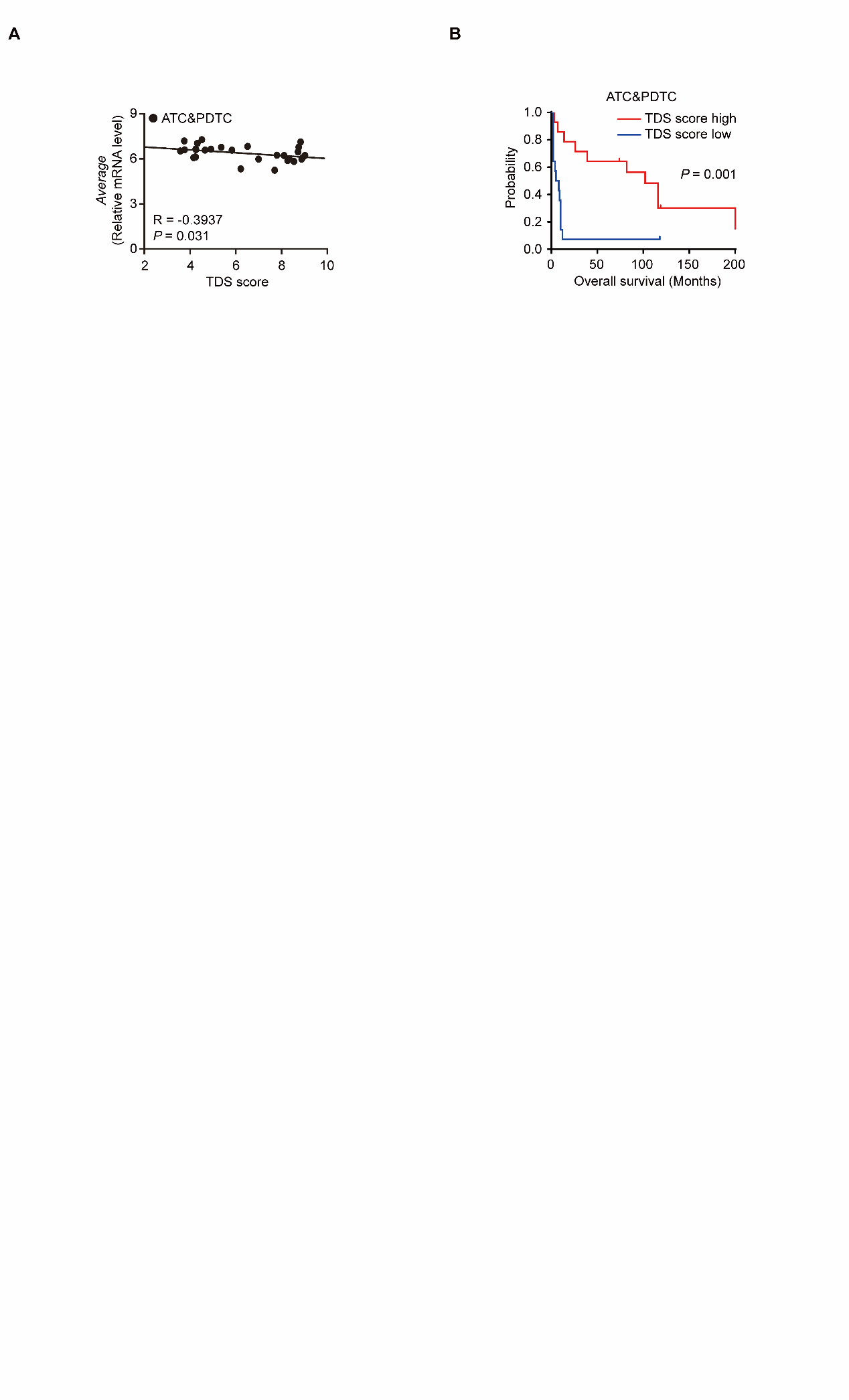


**Supplemantary Fig. 10 TDS low group shows poor prognosis in aggressive thyroid cancer.** **A** Graph exhibits negative correlation between the average of total eleven one carbon metabolism genes (*y*-axis) and TDS score (*x*-axis) in ATC and PDTC patients. The black lines indicate simple linear regression. **B** Kaplan-Meier survival curve shows the overall survival in ATC and PDTC patients separated by TDS score high (n=15) and low (n=15) groups. The GSE76039 consists of PDTC patients (n=17) and ATC patients (n=18).

**Supplementary Table. 1 Sequences of primers used in real-time RT-PCR.**

| Gene | Direction (5’ to 3’) | Sequences |
| --- | --- | --- |
| *PHGDH* | F | CTG CGG AAA GTG CTC ATC AGT |
|  | R | TGG CAG AGC GAA CAA TAA GGC |
| *SHMT2* | F | ATG TCT ATG CCC TAT AAG CTC AAC CC |
|  | R | GCC GGA AAA GTC GAG CAG T |
| *MTHFD2* | F | AGG ACG AAT GTG TTT GGA TCA G |
|  | R | GGA ATG CCA GTT CGC TTG ATT A |
| *MTHFD1L* | F | CTG CCT TCA AGC CGG TTC TT |
|  | R | TTT CCT GCA TCA AGT TGT CGT |
| *PSAT1* | F | ACA GGA GCT TGG TCA GCT AAG |
|  | R | CAT GCA CCG TCT CAT TTG CG |
| *SHMT1* | F | AGG AAA GGA GTG AAA AGT GTG GAT |
|  | R | GAC ACC AGT GTC GCT CTG GAT CTG |
| *MTHFD1* | F | AGG ATG TGG ATG GAT TGA CTA GC |
|  | R | CCC TTA GGC GTA CAA GGA ATG |
| *m36B4* | F | CGT CCT CGT TGG AGT GAC A |
|  | R | CGG TGC GTC AGG GAT TG |

**Supplementary Table. 2 Quality control and percent of doublet for single cell analysis.**

| Samples | GSM | Percent MT  (<) | nFeature_RNA  (>) | nFeature_RNA  (<) | nCount_RNA  (>) | Doublet  (%) |
| --- | --- | --- | --- | --- | --- | --- |
| ATC_1 | 4476491 | 10 | 500 | 6000 | 1000 | 1.0 |
| ATC_2 | 4476492 | 10 | 300 | 4500 | 1000 | 2.5 |
| ATC_3 | 4476493 | 15 | 300 | 6000 | 1000 | 1.3 |
| ATC_4 | 4476494 | 10 | 500 | 6000 | 1000 | 1.0 |
| ATC_5 | 4476495 | 10 | 500 | 6500 | 1000 | 2.5 |
| PTC_1 | 5585102 | 10 | 200 | 3500 | 1000 | 2.5 |
| PTC_2 | 5585104 | 10 | 200 | 1700 | 500 | 2.5 |
| PTC_3 | 5585107 | 10 | 200 | 2000 | 500 | 3.5 |
| PTC_4 | 5585112 | 10 | 200 | 3000 | 500 | 2.0 |
| PTC_5 | 5585117 | 10 | 200 | 4000 | 500 | 4.5 |
| PTC_6 | 5585119 | 10 | 500 | 4000 | 500 | 4.5 |
| PTC_7 | 5585121 | 10 | 200 | 2000 | 500 | 8.0 |
