# Supplementary material for: Co-inhibition of glutaminolysis and one-carbon metabolism promotes ROS accumulation leading to enhancement of chemotherapeutic efficacy in anaplastic thyroid cancer
Source: Cell Death Dis. 2023 Aug 12;14(8):515. doi: 10.1038/s41419-023-06041-2 (PMC10423221; doi:10.1038/s41419-023-06041-2)

**Full and uncropped figures­**

**Fig. 2I**


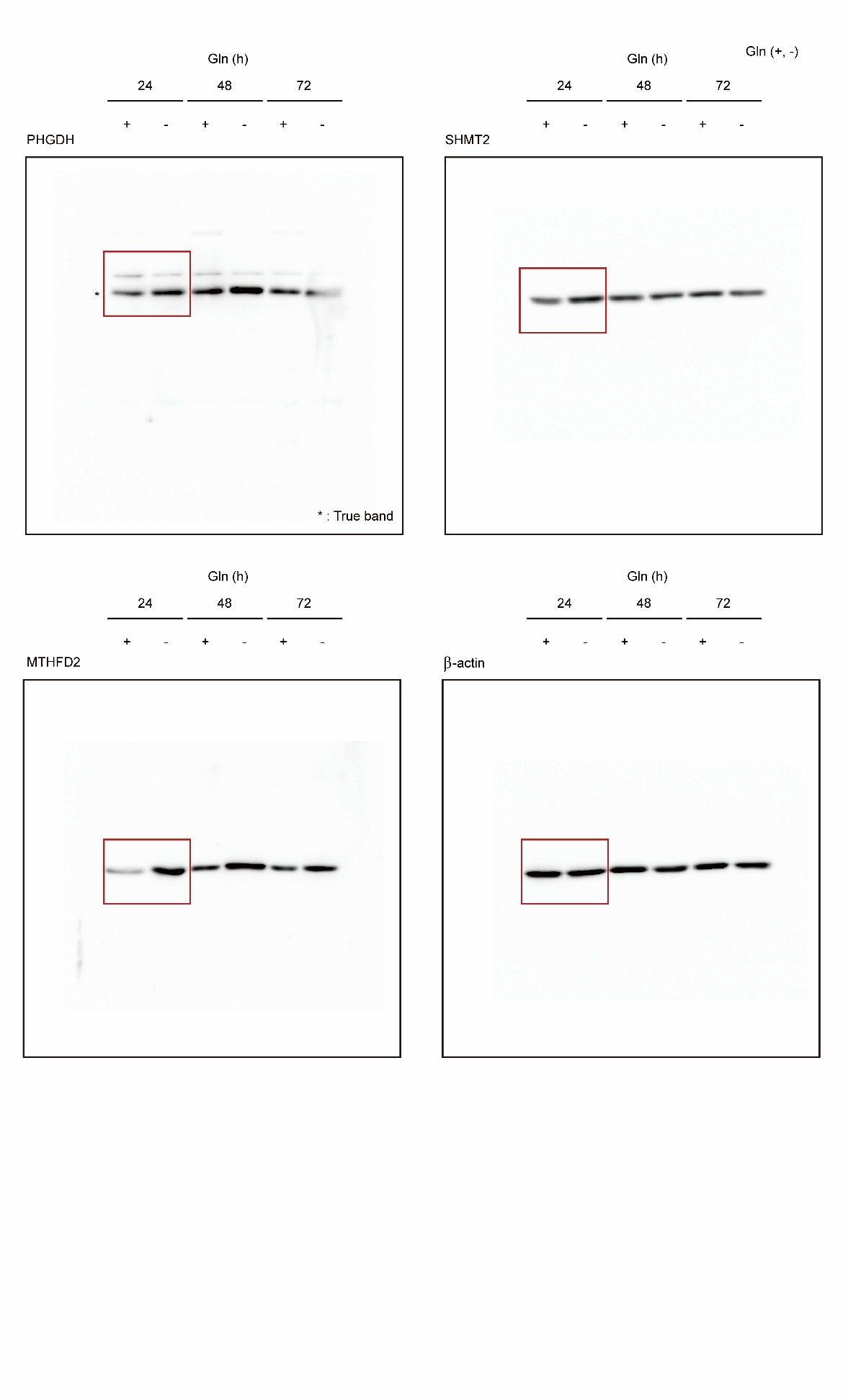


**Fig. 3H**


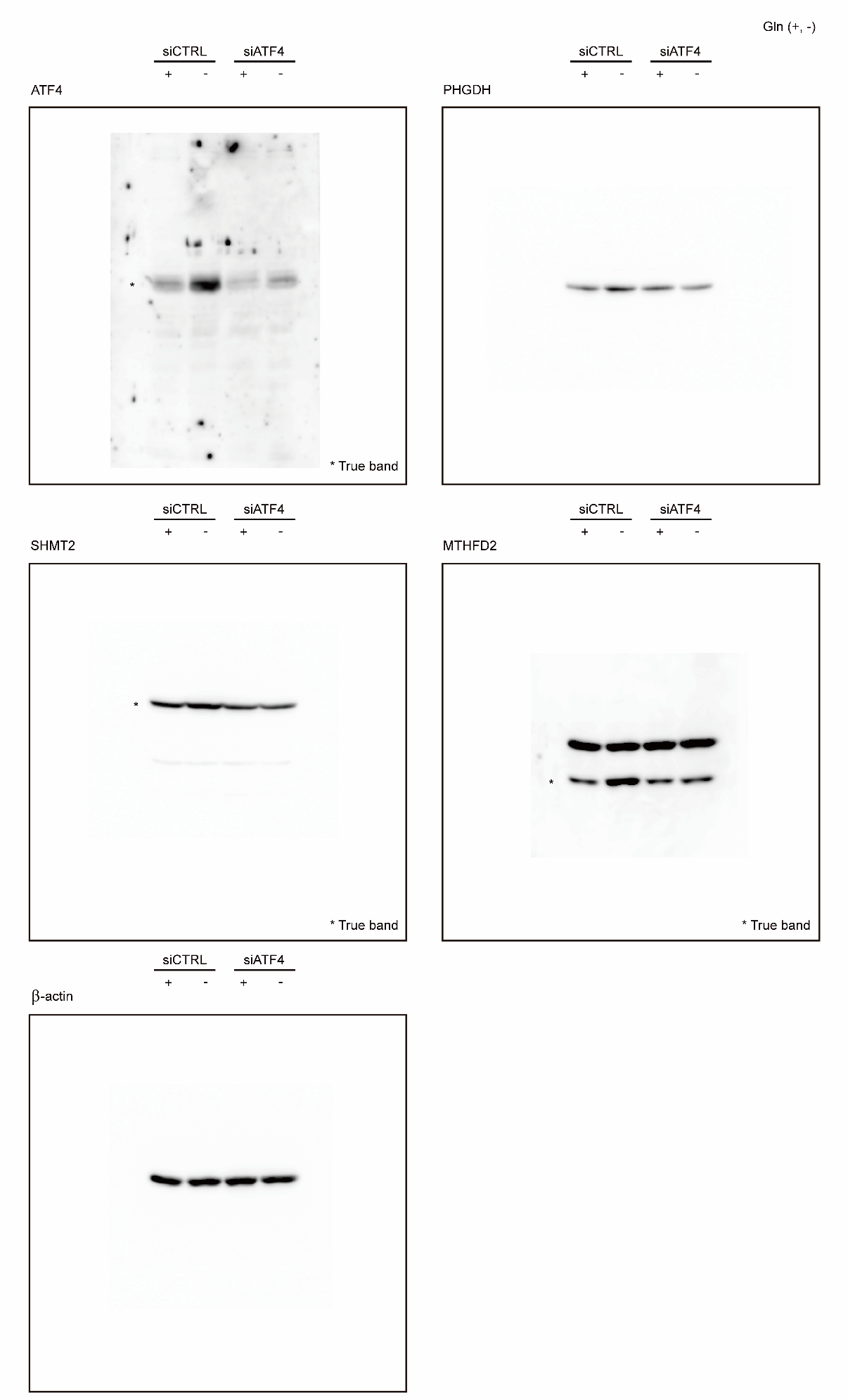


**Supplemantary Fig. 1C**


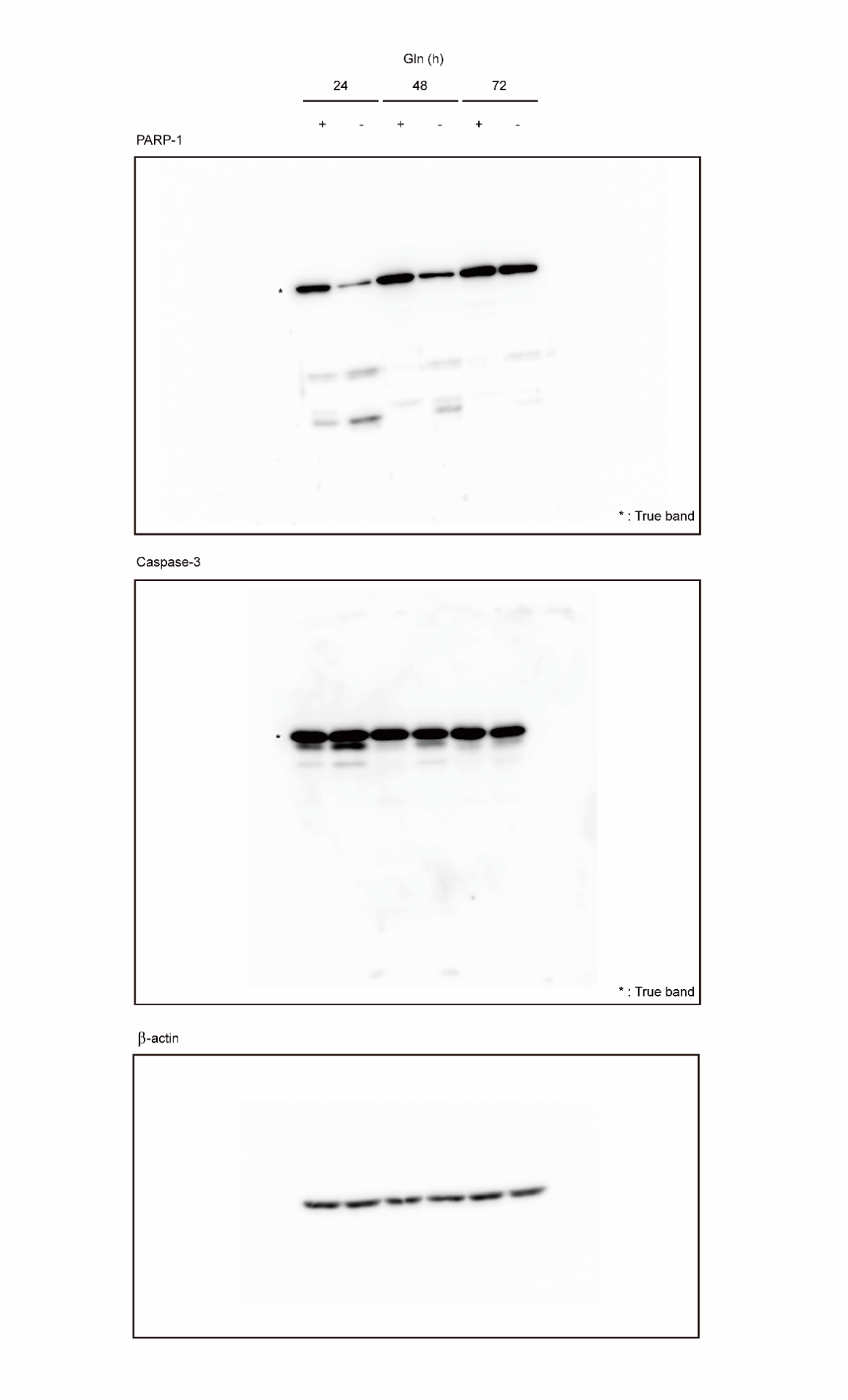


**Supplemantary Fig. 1C**


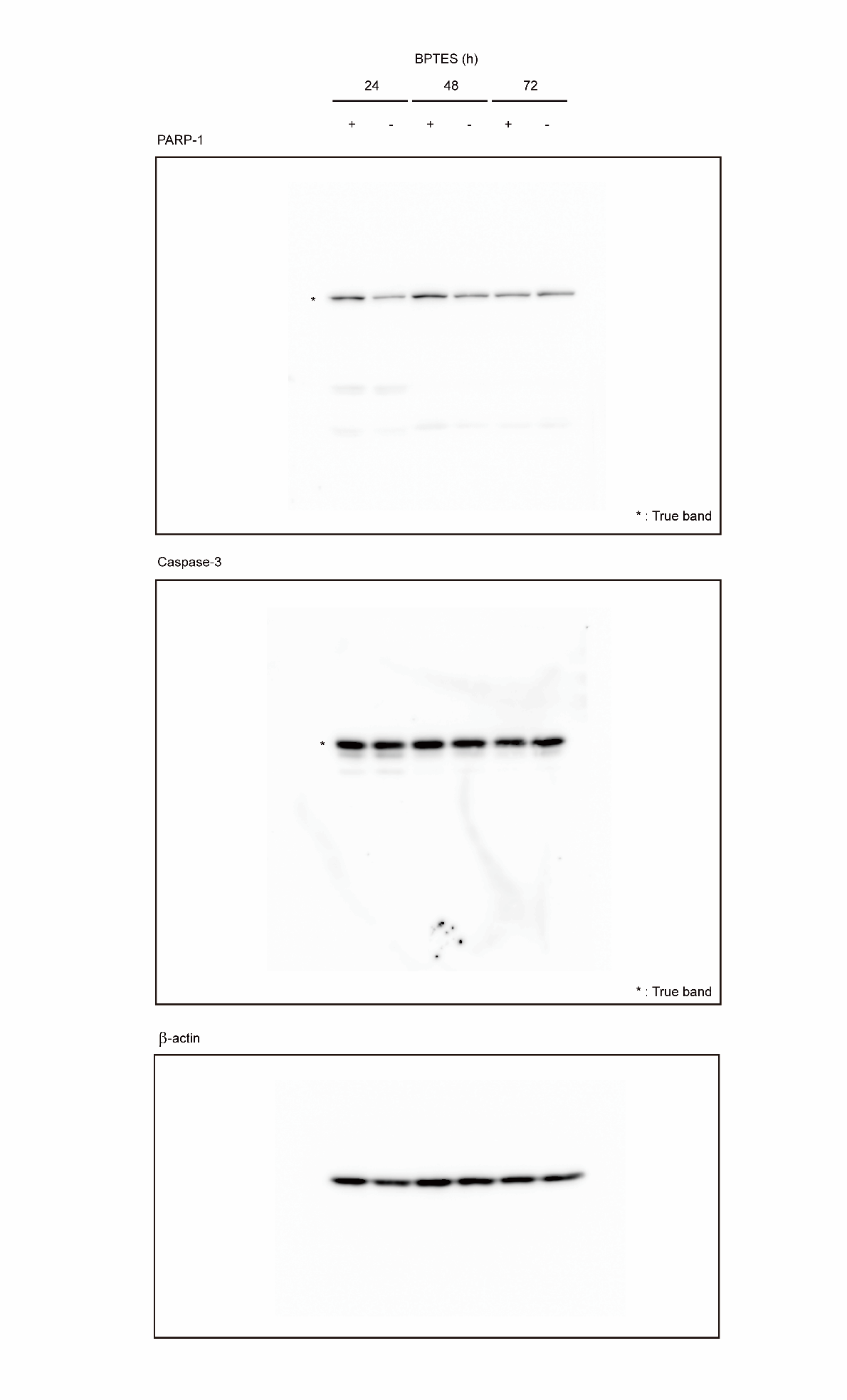


**Supplemantary Fig. 3**


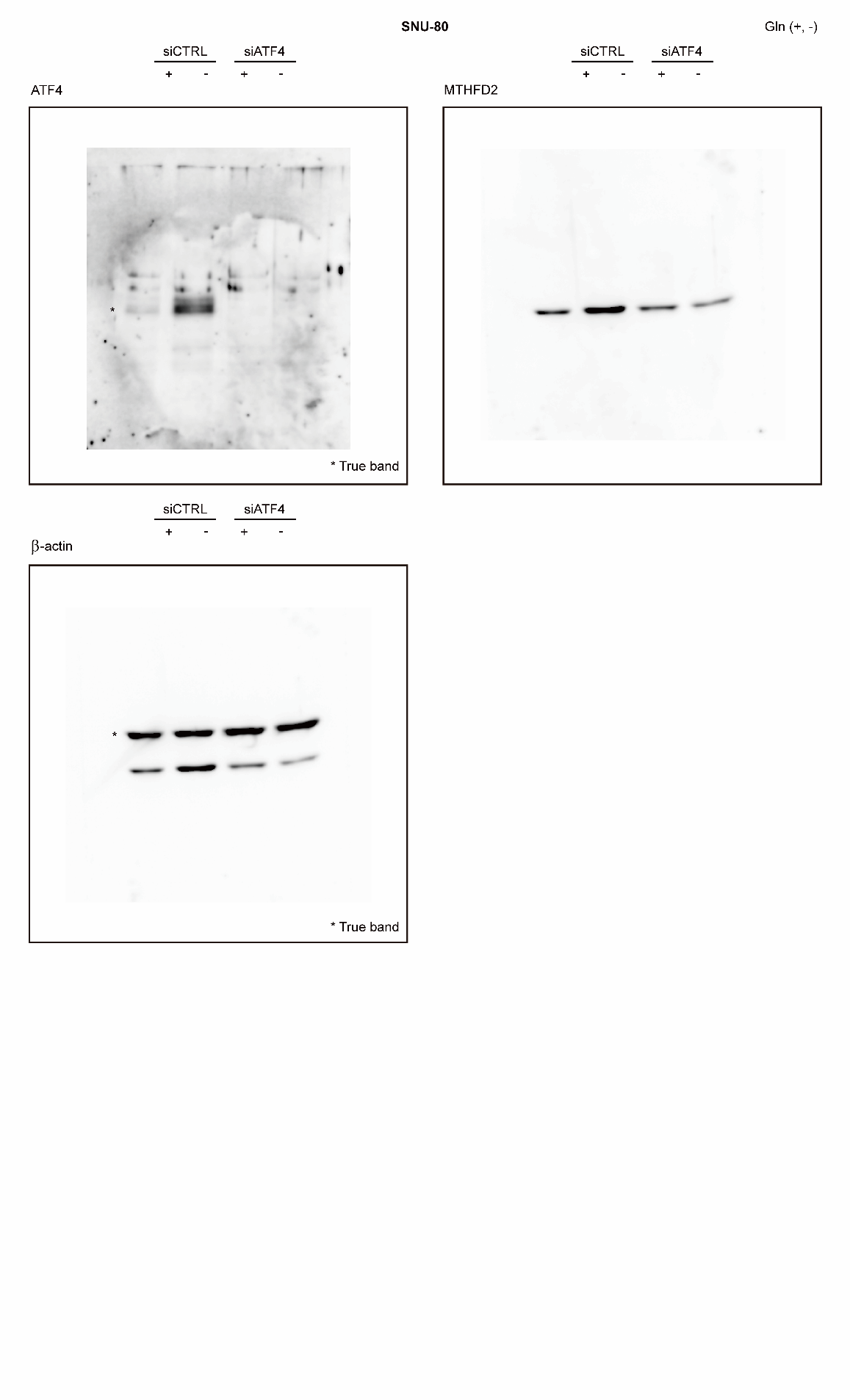

Supplement: Supplementary file 2 — Original data files [file 41419_2023_6041_MOESM2_ESM.docx]
